# Green synthesis of AgNP–ligand complexes and their toxicological effects on *Nilaparvata lugens*

**DOI:** 10.1186/s12951-021-01068-z

**Published:** 2021-10-13

**Authors:** Hatem Fouad, Guiying Yang, Ahmed A. El-Sayed, Guofeng Mao, Diab Khalafallah, Mahmoud Saad, Hassan Ga’al, Ezzeldin Ibrahim, Jianchu Mo

**Affiliations:** 1 Ministry of Agriculture, Key Laboratory of Molecular Biology of Crop Pathogens and Insect Pests, Key Laboratory of Biology of Crop Pathogens and Insects of Zhejiang Province, Institute of Insect Sciences, College of Agriculture and Biotechnology, Zhejiang University, Yuhangtang Road 866, Hangzhou, Zhejiang 310058 People’s Republic of China; 2 Department of Field Crop Pests, Plant Protection Research Institute, Agricultural Research Centre 12622, Dokki, Cairo Egypt; 3 Photochemistry Department, National Research Center, Dokki, Giza Egypt; 4 State Key Laboratory of Silicon Material, School of Materials Science and Engineering, Zhejiang University, Hangzhou, China; 5 State Key Laboratory of Rice Biology and Ministry of Agriculture Key Lab of Molecular Biology of Crop Pathogens and Insects, Institute of Biotechnology, Zhejiang University, Hangzhou, 310058 China

**Keywords:** Nanotechnology, Organic ligands, Silver nanoparticles, Insecticidal effect, *Nilaparvata lugens*

## Abstract

**Background:**

Despite developments in nanotechnology for use in the pharmaceutical field, there is still a need for implementation of this technology in agrochemistry. In this study, silver nanoparticles (AgNPs) were successfully prepared by a facile and an eco-friendly route using two different ligands, 2ʹ-amino-1,1ʹ:4ʹ,1″-terphenyl-3,3″,5,5″-tetracarboxylic acid (H_4_L) and 1,3,6,8-tetrakis (*p*-benzoic acid)-pyrene (TBAPy), as reducing agents. The physiochemical properties of the as-obtained AgNPs were characterized by scanning electron microscopy (SEM), energy-dispersive X-ray (EDX), X-ray diffraction (XRD) and transmission electron microscopy (TEM). The toxicity of H_4_L–AgNP and TBAPy–AgNP against the brown planthopper (BPH, *Nilaparvata lugens*) was also measured.

**Results:**

SEM and TEM analyses demonstrated the formation of quasi-spherical AgNP structures in the presence of H_4_L and TBAPy. Insecticidal assays showed that TBAPy is less effective against *N. lugens*, with a median lethal concentration (LC_50_) of 810 mg/L, while the toxicity of H_4_L increased and their LC_50_ reached 786 mg/L 168 h posttreatment at a high concentration of 2000 mg/L. H_4_L–AgNPs were also highly toxic at a low concentration of 20 mg/L, with LC_50_ =  ~ 3.9 mg/L 168 h posttreatment, while TBAPy–AgNPs exhibited less toxicity at the same concentration, with LC_50_ =  ~ 4.6 mg/L.

**Conclusions:**

These results suggest that the synthesized AgNPs using the two ligands may be a safe and cheaper method compared with chemical insecticides for protection of rice plants from pests and has potential as an effective insecticide in the *N. lugens* pest management program.

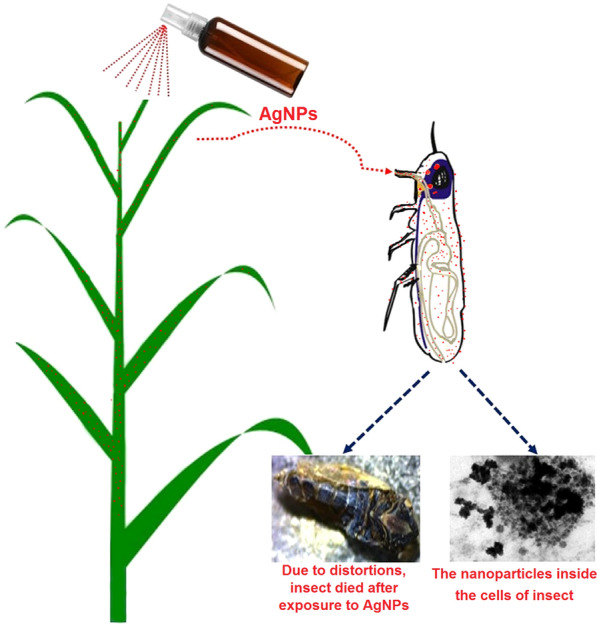

**Supplementary Information:**

The online version contains supplementary material available at 10.1186/s12951-021-01068-z.

## Background

Nanotechnology has been explored to determine various physical and chemical ways to use nanosized silver (Ag) particles [1, 2]. Noble metal nanoparticles (NPs) are widely used in electrochemical, electrodiagnostic, and bioelectrochemical applications because of their significant electroreactant activity due to their relatively high surface-area-to-volume ratio and interface-subjugated properties [3, 4]. The medical use of NPs is attracting vast interest because of their low toxicity in different nontarget organs or organisms [5, 6]. Noble metal NPs (i.e., Ag, Pt, Au, and Pd) are studied due to their low toxicity and low risk on the environment [7, 8]. In particular, AgNPs have attracted great attention in the field of biology because of their exceptional and tunable surface plasmon resonance (SPR) [9, 10]. Therefore, AgNPs have promising potential in bioscience, originating from their low ecotoxicological characterization [11, 12].

The brown planthopper (BPH) *Nilaparvata lugens* (Stål; Homoptera: Delphacidae) is an economically significant insect pest of rice plants in Asia [13]. BPH is distributed throughout many countries, such as Australia, China, Bangladesh, Cambodia, Fiji, India, Indonesia, and Japan. BPH causes damage to rice plants directly through feeding, and the plants turn yellow and rapidly dry up; this condition is called “hopper burn.” In addition, BPH causes extensive harm to rice plants by transmitting two viruses, rice ragged stunt virus and rice grassy stunt virus [14]. Because of its high adaptive ability to changing social practices, high reproductive potential, and long-distance migrating capability, insecticides are essential and applied widely to control this monophagous insect throughout Asia [15]. However, the extensive use of insecticides to control this pest over many years has led to resistance to most of classes of chemical insecticides (organochlorines, organophosphates, carbamates, and pyrethroids). In addition, BPH has distinct biological and behavioral attributes, for example, short up-growth time, high fertility, and dispersal ability [16–18]. The increase in insects resistant to chemical insecticides and toxicity concerns have motivated researchers toward the development of new insecticides based on AgNPs [11, 19, 20]. The organic ligand 2’-amino-1,1ʹ:4ʹ,1″-terphenyl-3,3″,5,5″-tetracarboxylic acid (H_4_L) was first reported by Bharadwaj et al. [21] to synthesize non-interpenetrated porous metal–organic frameworks (PMOFs) containing paddle-wheel secondary bonding units (SBUs). Pyrene and its derivatives have been widely investigated as fluorescence probes in many applications. For example, pyrene-labeled oligonucleotides have been used to study DNA hybridization [22, 23], and pyrene-labeled lipids have been developed to examine the depth-dependent quenching of fluorescence in lipid bilayers [24–26]. Recently, pyrene was used as a ligand to synthesize a mesoporous MOF and a catalytically active iridium pincer complex [27, 28].

Exploring an effective and versatile synthesis route to fabricate noble metal NPs without any toxic chemicals is still a challenge but desirable. In this study, we developed an efficient liquid-phase reduction method to produce a Ag colloidal suspension using two organic compounds in the presence of silver nitrate (AgNO_3_) as a metallic precursor. Such an approach could guarantee suspended AgNPs through the solution without any additives and toxic substances. Transmission electron microscopy (TEM) micrographs confirmed the formation of an Ag^0^ nanocluster in the H_4_L and 1,3,6,8-tetrakis (*p*-benzoic acid)-pyrene (TBAPy) ligands. The prepared H_4_L–AgNPs and TBAPy–AgNPs exhibited remarkable performance as insecticidal agents against BPH. Furthermore, the effect of treatment on enzyme levels in the insect system was explored and the action mechanism addressed as well. To the best of our knowledge, this is the first report on the synthesis, characterization, and insecticidal potential of AgNPs against BPH using the two organic ligands H_4_L and TBAPy.

## Materials and methods

### Chemicals

AgNO_3_, ethanol, tertbutanol, nitrobenzene, 4-methoxycaronylphenyl boronic acid, and tetrakis (triphenylphosphine) palladium were purchased from Sigma-Aldrich (St. Louis, MO, USA). 3,5-Dimethylboronic acid, KMnO_4,_ sodium hydroxide, H_2_SO_4_, 2,5-dibromoaniline, dimethylformamide (DMF), hydrochloric acid, sodium carbonate, palladium acetate, pyrene, nitrobenzene, bromine solution, nitrobenzene, ethyl acetate, n-hexane, potassium tribasic phosphate anhydrous, 1,4-dioxane, chloroform (THE), magnesium sulfate anhydrous, dimethyl sulfoxide, and potassium hydroxide (KOH) were purchased from Aladdin Biochemical Technology Co., Ltd. (Shanghai, China). Proton nuclear magnetic resonance (^1^H NMR) spectra were recorded using a Varian EM-390 spectrometer (USA) (400 MHz) with trimethylsilyl chloride (TMS) as an internal reference, and the chemical shift (*δ*) was expressed in parts per million (ppm). Mass spectra were recorded on a Kratos MS (75 eV) instrument (Kyoto, Japan). All reactions were monitored by thin-layer chromatography conducted on 0.2 mm silica gel 60F-254 (Merck) plates using UV light (245 and 365 nm) for detection. Acetylcholine iodide, 5,5-dithiobis-2-nitro benzoic acid, fast blue salt, α- and β-naphthyl acetate, Folin–Ciocalteu reagent were obtained from Sigma-Aldrich; β-nitrophenyl phosphate from Biotech (Shanghai, China); and all other chemicals and reagents, of the highest analytical grade, from local companies.

### Synthesis of AgNPs with H_4_L and TBAPy

AgNPs were prepared by direct reduction of the Ag source (i.e., AgNO_3_) in the presence of H_4_L and TBAPy. AgNO_3_ solution (1, 3, and 5 mM) was prepared in 150 mL of Milli-Q water and kept at 60 °C in a water bath for 10 min. Then, a defined amount of H_4_L or TBAPy dissolved in 2 mL of dimethyl sulfoxide was mixed in with vigorous magnetic stirring at 70 °C for 2 h before cooling down to room temperature. The reaction between ligands and Ag persisted under stirring in the dark at room temperature, and the formation of a Ag^0^ colloidal suspension was observed by optical color change (a reddish-brown color with H_4_L and a yellowish-black color with TBAPy). Additionally, the reduction of Ag ions to AgNPs was confirmed by sampling an aliquot of the reaction solution (4 mL) and analyzing it with a UV–Vis spectrophotometer. The H_4_L– and TBAPy–AgNPs were centrifuged at 10,000 rpm, and the colloidal suspension was collected and maintained in a vacuum oven at 120 °C for 2 h before further characterization.

### Characterization of synthesized AgNPs

#### UV–Vis spectral analysis of AgNPs

The UV–Vis patterns of the AgNP samples were recorded using 1 mL of AgNPs diluted with 3 mL of double-distilled water on a UV-2550 spectrophotometer (Shimadzu, Japan) at a wavelength of 200–800 nm with a resolution of 1 nm [29].

#### FT-IR analysis of AgNPs

Spectra of the dry powder of the AgNP samples were recorded under identical conditions at 500–4000 cm^−1^ with an amplitude of 4 cm^−1^ using Fourier transform infrared spectrometer (FT-IR, Vector 22; Bruker Corporation, Germany). Before measurements, 1 mg of the AgNP sample was mixed with 300 mg of KBr, cast into circular pellets, and stabilized under reactive humidity.

#### EDX analysis of AgNPs

The surface of the as-synthesized AgNP samples was viewed by an energy-dispersive X-ray spectroscopy (EDX, SU-8010; Hitachi, Japan). A thin film of the prepared AgNP samples was deposited onto a well-cleaned silicon substrate and gently dried. Next, the samples were attached to a circular metallic stud by carbon tape and inserted into the vacuum chamber of the microscope using a cylindrical lever. Analyses were performed at an accelerating voltage of 3 kV.

#### TEM and XRD analyses of AgNPs

The morphologies and microstructures of the obtained H_4_L– and TBAPy–AgNPs were observed by TEM (JEOL-JEM-1230; JEOL, Japan) operated at an accelerating voltage of 200 kV. The samples were mixed with ethanol under gentle sonication to disperse the powder into the solution well. A drop of the suspension was pipetted onto a copper grid and then dried at room temperature. The phase purity and crystalline structure of the as-synthesized compounds were measured by wide-angle X-ray diffraction (XRD) using a Siemens X-ray diffractometer (Siemens, Germany). The XRD patterns were collected with a monochromated CuKα radiation source at a potential of 40 kV and a current of 30 mA [30].

#### 3Dynamic light scattering (DLS) and Zeta potential

The average particle size and size distribution of AgNPs were evaluated using the particle size analyzer system (Zeta sizer, Malvern Instruments Ltd., USA), with the same device, the zeta potential of AgNPs was measured to determine the stability of nanoparticles.

#### Insect rearing

BPH adults were initially gathered from rice fields in Hangzhou, Zhejiang Province, China, and reared on a susceptible variety of rice (TN1) in net cages at 26 ± 2 °C in a 14:10 h light:day (L:D) cycle at 70% − 80% relative humidity in the Laboratory of Urban Entomology, Institute of Insect Sciences, Zhejiang University, China. All tested insects were preserved on the same rice variety. Both sexes of BPH were collected from the population (20 individuals/replicate) and used in the experiment.

#### Toxicity tests of two AgNP–ligand complexes against the brown planthopper

The standard methods to determine the lethal concentration of H_4_L– and TBAPy–AgNPs on BPH adults were followed. Before defining the final doses of compounds, a preliminary test was performed to determine the dosage range of an insecticide with different levels of mortality before conducting toxicity tests. The concentrations of all samples were 500, 1000, 1500, and 2000 mg/L prepared in a 100 mL beaker Borosil (Mumbai, India) using deionized water as a solvent for the two ligands without Ag (positive control). For toxicity tests, H_4_L– and TBAPy–AgNP colloids were diluted using double-distilled water as a solvent to the desired concentrations (5, 10, 15, and 20 mg/L). Dimethyl sulfoxide was used as a negative control. Next, 20 BPH adults per replicate were released on rice plants in plastic cups (500 mL) and covered with water-impregnated cotton. After spraying the prepared compounds, all cups were covered with muslin cloth held in place with rubber bands to prevent the insects from escaping during the experiment. Each toxicity test, including a set of the control group (distilled water), was tested with four replicates of four concentrations of each compound. The plastic cups with treated insects were put into an incubator at 26 ± 1 °C in a 14:10 h L:D photoperiod. Mortality was recorded at 24 h intervals up to 10 days after spraying. Moribund insects were also considered for determining mortality after gentle prodding with a fine brush.

### Mechanisms of action of AgNPs on *N. lugens*

#### Preparation of whole-body homogenates

The treatment and control BPH groups (15 adult individuals per group) were pooled and homogenized in Eppendorf tubes (held in crushed ice) using a Teflon hand homogenizer in 1 mL of 0.9% saline for ultimate estimation of total proteins, acetylcholine, esterases, and phosphatases action. The whole-body BPH homogenates were centrifuged at 12,000 × *g* and 4 °C for 10 min, and the clear supernatants were used for biochemical examination. The solution for homogenization and glassware were kept at 4 °C before use, and the homogenates were hung on ice before further investigation.

#### Determination of protein concentration

The proteins in BPH whole-body homogenates were first precipitated by 80% ethanol [31], and the protein concentration was estimated using the Lowry method [32].

#### Acetylcholinesterase assays

With slight modifications, acetylcholinesterase activity in the BPH whole-body homogenates was spectrophotometrically measured using acetylcholine iodide as a substrate [33]. Each aliquot of the homogenate (200 µL) was mixed successively with 200 µL of sodium phosphate buffer (100 mM, pH 7.5), 50 µL of 10 mM 5,5′-dithiobis (2-nitrobenzoic acid) (DTNB) and 50 µL of 12.5 mM acetylcholine iodide. After incubation for 5 min at room temperature, the optical density of the samples was tested at 400 nm against a suitable reagent blank.

#### Esterase assay

Carboxylesterase activity in the whole-body BPH homogenates was determined using the Van Asperen method [34]. Briefly, 200 μL of control and treatment BPH homogenates was mixed with 2 mL of the α- or β-naphthyl acetate solution, and the reaction was extended for 30 min at room temperature. After incubation, 500 μL of fast blue sodium dodecyl sulfate (SDS) reagent was added (22.5 mg OF fast blue salt in 2.25 mL OF distilled water and 5% w/v SDS in 0.2 M phosphate buffer; pH 7.2), and the color was allowed to develop for 15 min at room temperature. The optical density of the samples was measured at 588 nm using a spectrophotometer against the respective reagent blank.

#### Phosphatase assay

The levels of acid and alkaline phosphatases in the whole-body BPH homogenates were measured using the Asakura method with slight modifications [35]. Acid phosphatase activity was estimated by mixing 100 μL of insect homogenate with 400 μL of 50 mM sodium acetate buffer (pH 4.0) and 500 μL of 15 mM β-nitrophenyl phosphate. Alkaline phosphatase activity was estimated by mixing 100 μL of BPH homogenate with 400 μL of 50 mM Tris–HCl buffer (pH 8.0) and 500 μL of 15 mM p-nitrophenyl phosphate. After incubation for 15 min at 37 °C in a water bath, the enzymatic reaction was stopped by adding 100 μL of 0.5 N NaOH solution and centrifuged at 4000 × *g* for 5 min. Absorbance of the resulting clear supernatant from each sample was checked at 440 nm against the appropriate reagent blank.

### Determination of AgNP content in the brown planthopper by TEM

The AgNP accumulation in the adult BPH body was evaluated by TEM [36]. Briefly, midgut extracts of AgNP treatment (LC_30_) and control groups were collected, anesthetized, and fixed in 2.5% glutaraldehyde in 0.1 M phosphate buffer (pH 7.2) for 48 h at 4 °C. The samples were washed several times with phosphate-buffered saline (PBS) for 15–20 min each time, then postfixed in 2% aqueous osmium tetroxide for 1–2 h, washed again with distilled water four to six times for 30–45 min each time, dehydrated in a series of alcohols, and embedded in spur resin. The samples were incubated at 80 °C overnight for complete polymerization. Ultrathin Sects. (50–70 nm) were cut with a glass knife on an ultramicrotome (LEICA EM UC7 ultratome) and then mounted on copper grids. The sections were stained with uranyl acetate and alkaline lead citrate for 5–10 min, and the samples were characterized with TEM (Model H-7650 TEM; Hitachi-Japan).

### Histological analysis

Histological analysis of the BPH digestive system was performed using adult insects (treatment and control groups). Briefly, 20 BPH adults were exposed to a median lethal concentration (LC_50_) of H_4_L, TBAPy, H_4_L–AgNPs, and TBAPy–AgNPs for 5 days, as previously described [37] with a small modification. Briefly, the insects were fixed in 10% buffered formaldehyde for 48 h, dehydrated using a graded series of ethanol, and cleared with xylene solution. Next, they were embedded in paraffin blocks using melted paraffin at the embedding station. The paraffin blocks were cut into 5-µm-thick sections using a rotary microtome and stained with hematoxylin and eosin. The glass slides were examined for abnormalities under a light microscope.

### Data analysis

The mortality (%) of BPH treated with H_4_L– and TBAPy–AgNPs were subjected to probit analysis for calculating LC_50_ and LC_90_ with 95% confidence limits of lower and upper values [38]. For evaluating the biological variables observed in the experiments, one-way analysis of variance (ANOVA) and Duncan’s test were performed to assess the variation between both treatments. Chi-square values and all analyses were analyzed using CoStat ver. 6.311 (CoHort Software, Monterey, USA), and SPSS Statistics ver. 16.0 (SPSS Inc., Chicago, IL, USA).

## Results and discussion

### Synthesis of H_4_L and TBAPy

H_4_L and TBAPy are two organic ligands formed during the synthesis of the frameworks L_(Cu)_ and NU-1000 [39, 40]. However, this study is the first attempt to prepare a Ag colloidal suspension as an insecticide using these ligands. H_4_L was obtained from the chemical reaction of 3,5-dimethylphenylboronic acid to form benzene-3,5-dicarboxyelester-boronic acid [41], and the final product was obtained according as previously described with slight modifications [40]. The reaction by-products and compounds were checked by elemental analysis, such as ^1^H NMR (Additional file 1: Scheme S1). TBAPy was fabricated via the initial bromination of pyrene, followed by the reaction of tetrabromopyrene with 4-methoxycaronylphenyl boronic acid as previously described with some modifications (Additional file 1: Scheme S2; Figures S1, S2, and S3).

### Formation mechanism of H_4_L– and TBAPy–AgNPs

**Fig. 1:**
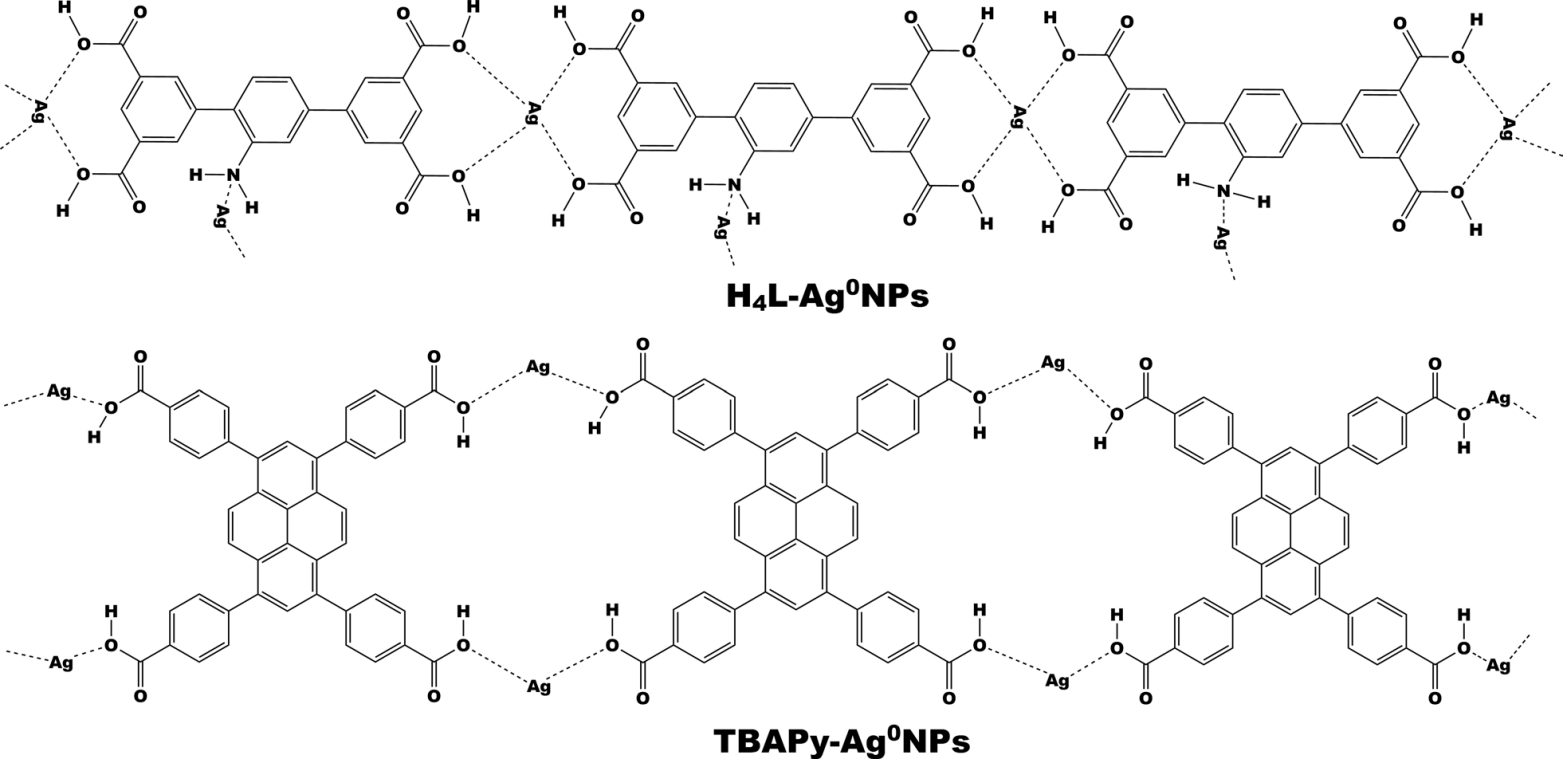
Hydrogen bonds form between organic ligands and silver after the process of reduction of Ag^+^ to Ag^0^ nanoparticles

H_4_L– and TBAPy–AgNPs were constructed by the direct recombination of Ag cations and ligand anions throughout the electrostatic attraction. The anchoring of Ag^+^ into the organic ligands to form H_4_L– and TBAPy–AgNPs via a simple liquid-phase reduction process was confirmed by different analyses, such as FT-IR, UV–Vis spectroscopy, XRD, TEM, and EDX, as described later. We proposed that the functional groups induced onto the surfaces of ligands facilitate the nucleation of Ag seeds at the start of the reaction and the primarily grown seeds tend to aggregate and recrystallize to form quasi-spherical AgNPs after a prolonged period of growth (Fig. 1). Furthermore, the induced functional groups might improve the binding between the metallic cations and the ligand species in the resulting complexes.

### Characterization of H_4_L– and TBAPy–AgNPs

#### UV–Vis spectroscopy

**Fig. 2:**
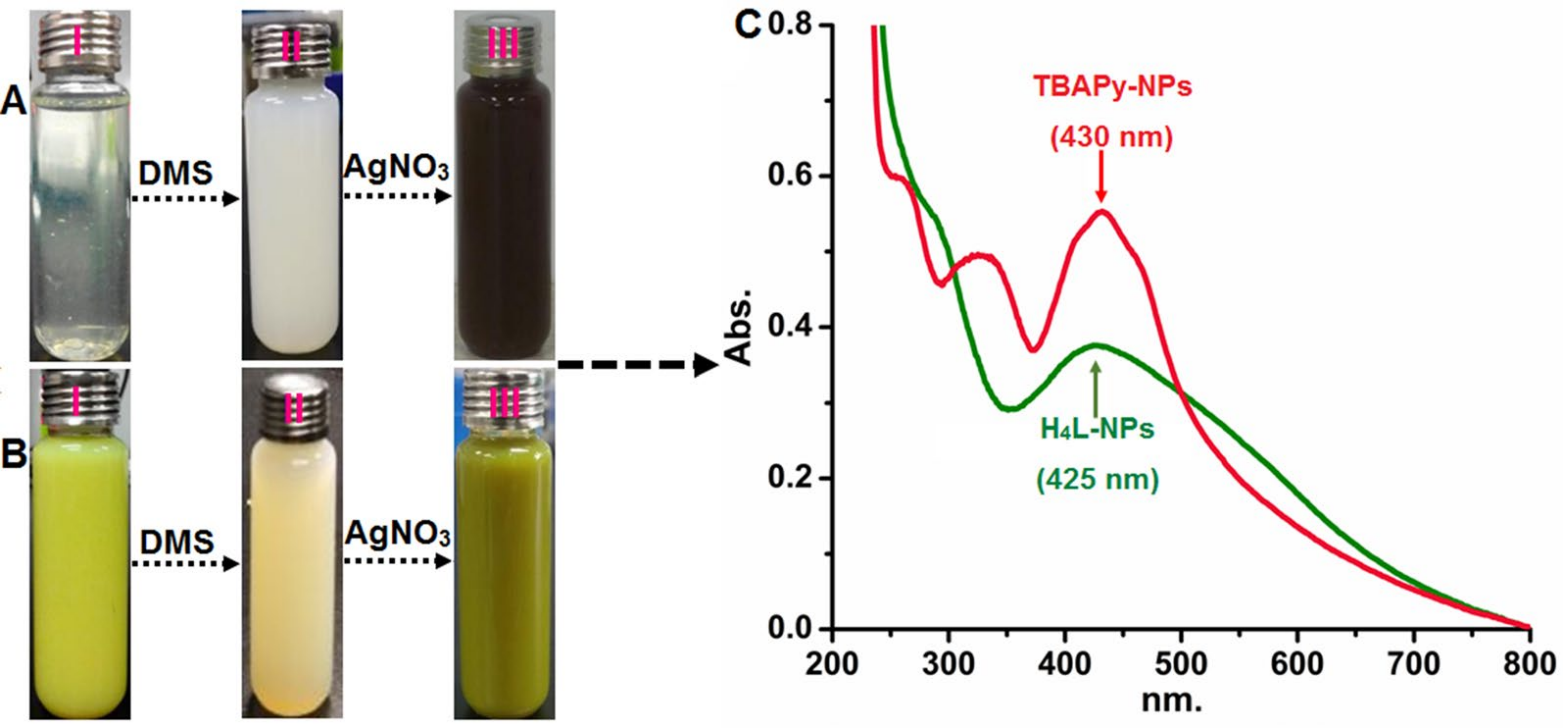
The preparation process of AgNPs, and variation of color changes in tested compounds. **A (I)** aqueous solution containing H_4_L and DMSO, (**II)** the solution containing AgNO_3_, (**III)** after the process of reduction of Ag^+^ to Ag^0^ with changed color to reddish-brown. **B (I)** aqueous solution containing TBAPy and DMSO, (**II)** the solution containing AgNO_3_, (**III)** after the process of reduction of Ag^+^ to Ag^0^ nanoparticles with changed color to yellowish-black. **C **UV–Vis absorption spectra of AgNPs synthesized using H_4_L and TBAPy

UV–Vis spectroscopy is a technique for verifying NP development in a solution. In this study, the integrated H_4_L– and TBAPy–AgNO_3_ systems underwent a chemical reduction process, and the change in color to a reddish-brown for H_4_L or yellowish-black for TBAPy indicated the formation of a Ag colloidal suspension (Fig. 2A, B). The obtained complexes were primarily observed by a UV spectrophotometer, and the recorded signals displayed major absorption peaks centered at 425 and 430 nm for H_4_L– and TBAPy–AgNPs, respectively, which is mainly indexed to generate AgNPs (Fig. 2C). Such characteristics match well with those observed for a single SPR at 405, 420, 448, and 450 nm [10, 11, 42]. The deviation of the SPR shoulder of anisotropic particles might be attributable to their shape and size [43, 44]. However, the control samples prepared without AgNO_3_ salt depicted an SPR peak at 230 nm originated from the organic matrices (Fig. 2C).

#### FT-IR analysis

**Fig. 3:**
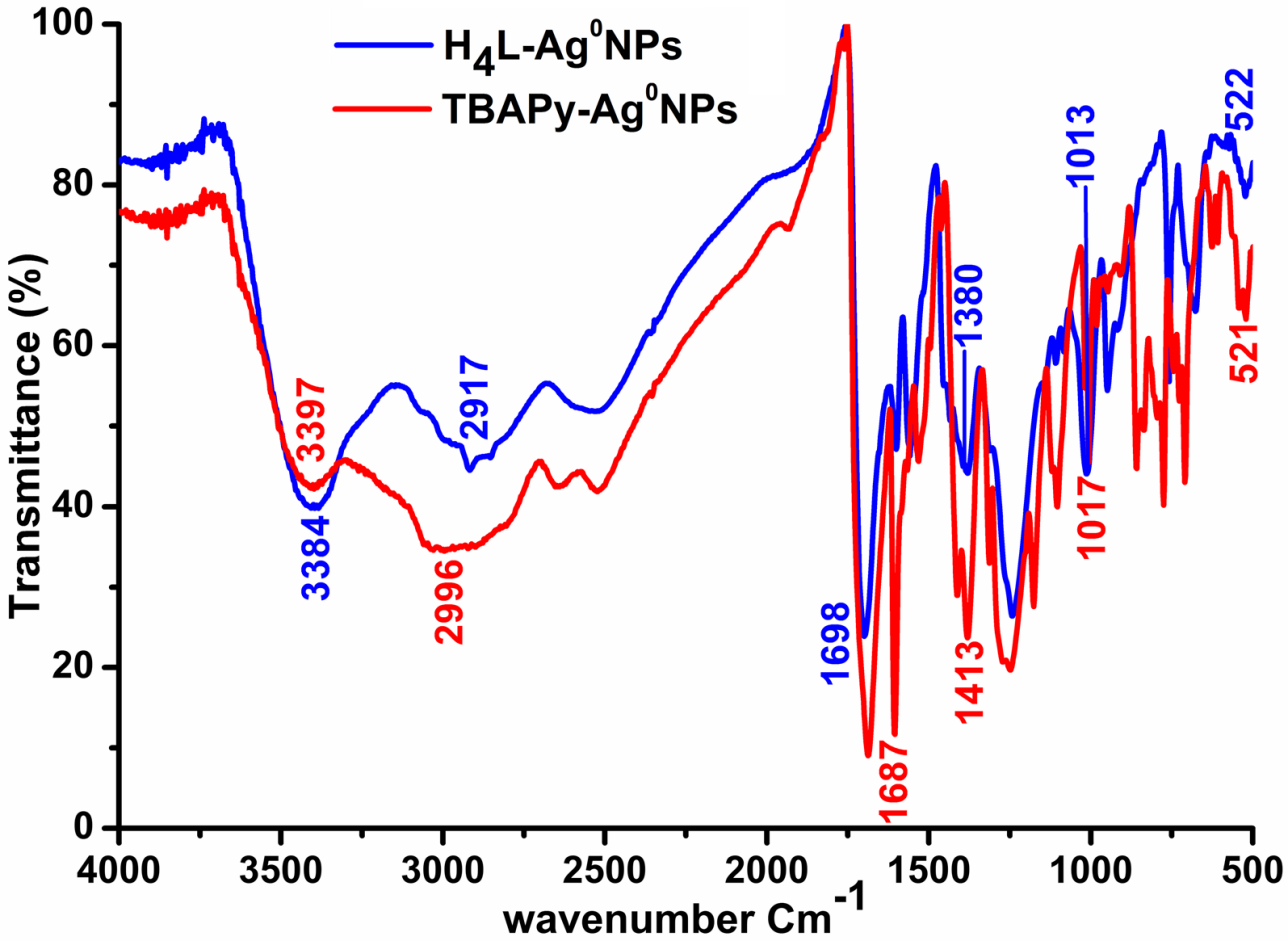
The FT-IR spectrum of silver nanoparticles synthesized using organic ligands H_4_L and TBAPy

FT-IR measurements were performed to obtain detailed information about the formation mechanism of AgNPs and functional groups. The responses of the as-designated H_4_L– and TBAPy–AgNPs were collected in the range from 500 to 4000 cm^−1^ (Fig. 3). The absorption bands of H_4_L–AgNPs were detected at 3384, 1698, 1380, 1013, and 522 cm^−1^ owing to the vibration of the chemical particles of H_4_L. For TBAPy–AgNPs, the characteristic bands were located at 3397, 1687, 1413, 1017, and 521 cm^−1^ within the same wavenumber window. The broad absorption peaks at 3384 and 3397 cm^−1^ for H_4_L– and TBAPy–AgNPs, respectively, can be attributed to the strong vibration modes of O–H stretching and bending, while the sharp characteristics peaks at 1698 and 1687 cm^−1^ for H_4_L– and TBAPy–AgNPs, respectively, are associated with the stretching vibration of carboxylic acid and some variable stretching and bending peaks that would be involved in the growth process of AgNPs [41]. In addition, the peak shift from 1380 to 1013 cm^−1^ for H_4_L–AgNPs and from 1413 to 1017 cm^−1^ for TBAPy–AgNPs is largely ascribed to the contribution of the –C–H and O–H bending vibration of aliphatic and aromatic C–H plane distortion vibrations of methyl, methylene, and methoxy groups during reduction. Such characteristic modes demonstrate the extended vibrational bands in charge of compounds such as flavonoids and terpenoids [44–46].

#### TEM, XRD, and EDX analyses of AgNP-based complexes

**Fig. 4:**
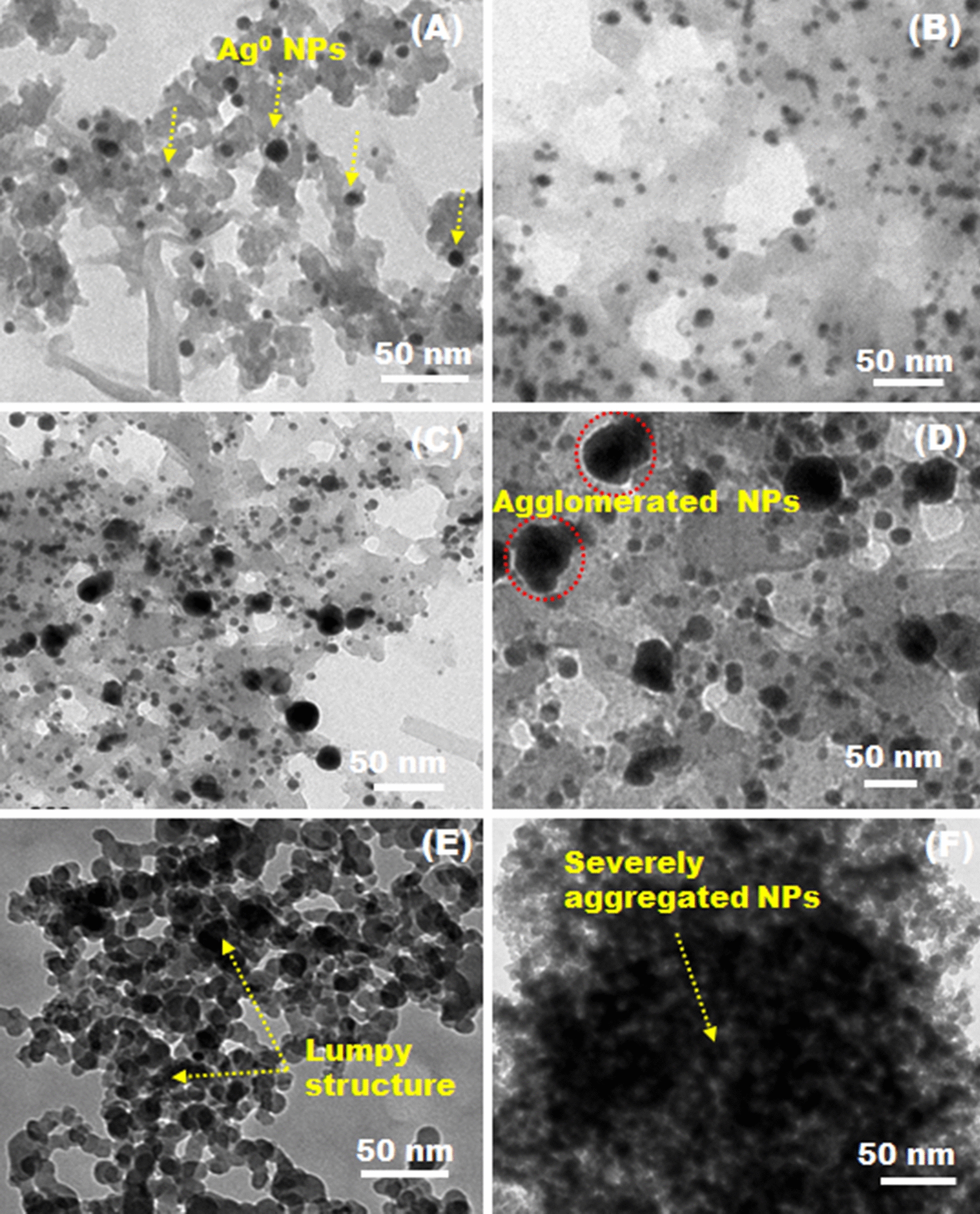
TEM micrographs of the as-prepared complexes. **A-C** H_4_L–AgNPs and **D-F** TBAPy–AgNPs. The observation illustrates the growth of particles for both complexes at different AgNO_3_ concentrations; **A **and **D** 1 mmol, **B** and **E** 3 mmol, and **C** and **F** 5 mmol

The microstructure and surface morphologies of the as-synthesized AgNPs were analyzed by TEM (Fig. 4). The TEM images of both H_4_L– and TBAPy–AgNPs at different concentrations of AgNO_3_ showed more microscopic structures of Ag^0^-intercalated organic ligands. The organic frameworks displayed thin films of biomolecules that are beneficial for attaining highly stable nanocrystals for prolonged durations. The samples exhibited quasi-spherical AgNPs anchored into the entire film of the underlying substrates etched into a porous structure. These AgNPs were firmly interconnected to the transparent networks, which is probably due to the electrostatic interaction between the metallic cations and the surface functional groups of the organic matrices, as well as interfacial interaction among NPs (Fig. 4). The dispersion of the Ag^0^ nanocluster formed by H_4_L was better than that TBAPy, suggesting a relatively low growth rate of NPs formed by H_4_L (Fig. 4C). The NPs formed by TBAPy revealed a lumpy morphology made up of agglomerated NPs (Fig. 4D-F). The Ag^0^ particles produced by both ligands appeared thicker with increasing AgNO_3_ concentration to 3 and 5 mM because of their high tendency to aggregate into larger clusters (Fig. 4 and Additional file 1: Figure S4), which is a result expected due to ultrafast dissociation of AgNO_3_ into water and thereby rapid growth kinetics. The darker areas are mainly associated with the agglomeration and condensed overlapping of the adjacent NPs because of thermodynamic kinetic changes. It seems reasonable that the reduction mode and growth mechanism of NPs evolves with the nature of the reducing agent and is strongly correlated with reaction conditions, such as time, temperature, and reactant concentration.

Accordingly, the reduction rate of AgNPs was fast at the initial stage of nucleation and crystallization. The freshly formed nanocrystals are thermodynamically unstable, owing to their high surface energies. Subsequently, these nanoclusters were guided to drive the following stage of self-assembly with extra generated nanocrystals into larger NPs by changing the corresponding interfacial energies. Moreover, the heating of the reaction solution with strong reflux may facilitate AgNP formation. We proposed that the variation in nanocrystal free energies might probably play a role in influencing the reaction kinetics and nucleation mechanism. It is worth mentioning that the architecture and geometry of the constructed NPs could significantly influence biological systems, particularly pest administration programs [47]. Accordingly, the obtained AgNPs are expected to play an important role in contributing to insecticidal assays.

**Fig. 5:**
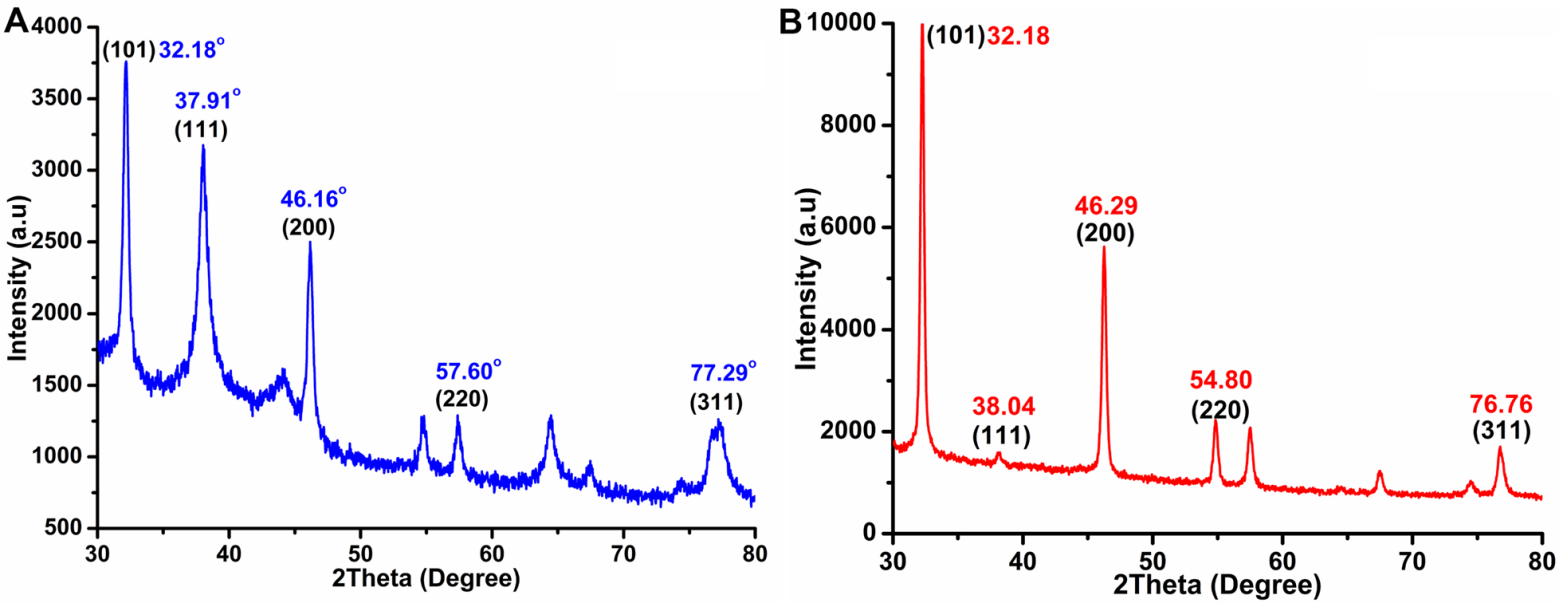
XRD patterns of synthesized silver nanoparticles **A** H_4_L–AgNP and **B** TBAPy–AgNP

The crystalline structure of the obtained AgNPs was studied by XRD analysis, as illustrated in Fig. 5. The H_4_L–AgNP spectrum (Fig. 5A) exhibited five diffraction peaks at 2*θ* of 32.18°, 37.91°, 46.16°, 57.60°, and 77.29°, indexed to [101], [111], [200], [220], and [311] planes of standard face-centered cubic AgNPs, respectively. The TBAPy–AgNP spectrum (Fig. 5B) displayed five major peaks at 2*θ* of 32.18°, 38.04°, 46.29°, 54.80°, and 76.76°, reflecting the [101], [111], [200], [220], and [311] diffractions of the cubic Ag phase, respectively. The slight change in the diffraction positions is probably due to the change in the chemical microstructure of the ligands. We believe that the induced strain can affect the character of nanocrystallites in the crystal structure. These results are consistent with those obtained for AgNPs prepared by using the cell-free supernatants of two bacteria *Bacillus amyloliquefaciens* and *B. subtilis* [11].

The composition and elemental analysis of both H_4_L– and TBAPy–AgNPs are illustrated Additional file 1: Figure S5. The mapping profiles of both complexes demonstrated that the bulk Ag represents the major component. The spectra of both H_4_L– and TBAPy–AgNPs (Additional file 1: Figures S6A and S6B) displayed a strong peak at 3 keV attributed to AgNPs and demonstrated the reduction of Ag^+^ to Ag^0^ with the assistance of H_4_L and TBAPy capping salts. Besides, Al, S, Si, Cl, and O probably originated from the silicon substrate used to prepare the samples prior to EDX analysis and the organic precursors used to prepare the two ligands. The corresponding cross-sectional compositional line profiles displaced that the Ag intensity is much stronger than that of other elements detected, which evidences the predominance of Ag in the NPs. These findings agree well with the reported EDX profiles and existing results in the literature [43, 48] and indicate the effectiveness of our ligands as environmentally friendly compounds to create Ag^0^-based complexes. Additionally, this may be an efficient strategy to prepare other noble metal NPs.

#### Nanoparticle size and stability of the nanoparticles

The average size of H_4_L-AgNP was found to be 7 nm to 75 nm, while the particle size of TBAPy-AgNP was found to be 10 nm to 85 nm (Additional file 1: Figure S7A, B). The zeta potential is usually utilized to know the constancy of colloidal systems. The zeta potential of nanoparticles was evaluated in methanol as dispersant. In this report, zeta potentials of H_4_L– and TBAPy–AgNPs were − 28.1 mV and − 27.2 mV, which denoted the stability of silver nanoparticles suspensions (Additional file 1: Figure S7C, D). Mostly, a suspension that displays an absolute zeta potential less than 20 mV is considered unstable and will cause precipitation of particles from solution, while the absolute zeta potential higher than 20 mV is stable [49, 50]. Guilger‑Casagrande et al. reported that all the samples of nanoparticles presented negative values with the AgNP-TS sample showing the highest electronegativity − 33.3 mV [51].

### Toxicity of AgNPs toward unsexed brown planthopper adults

**Fig. 6:**
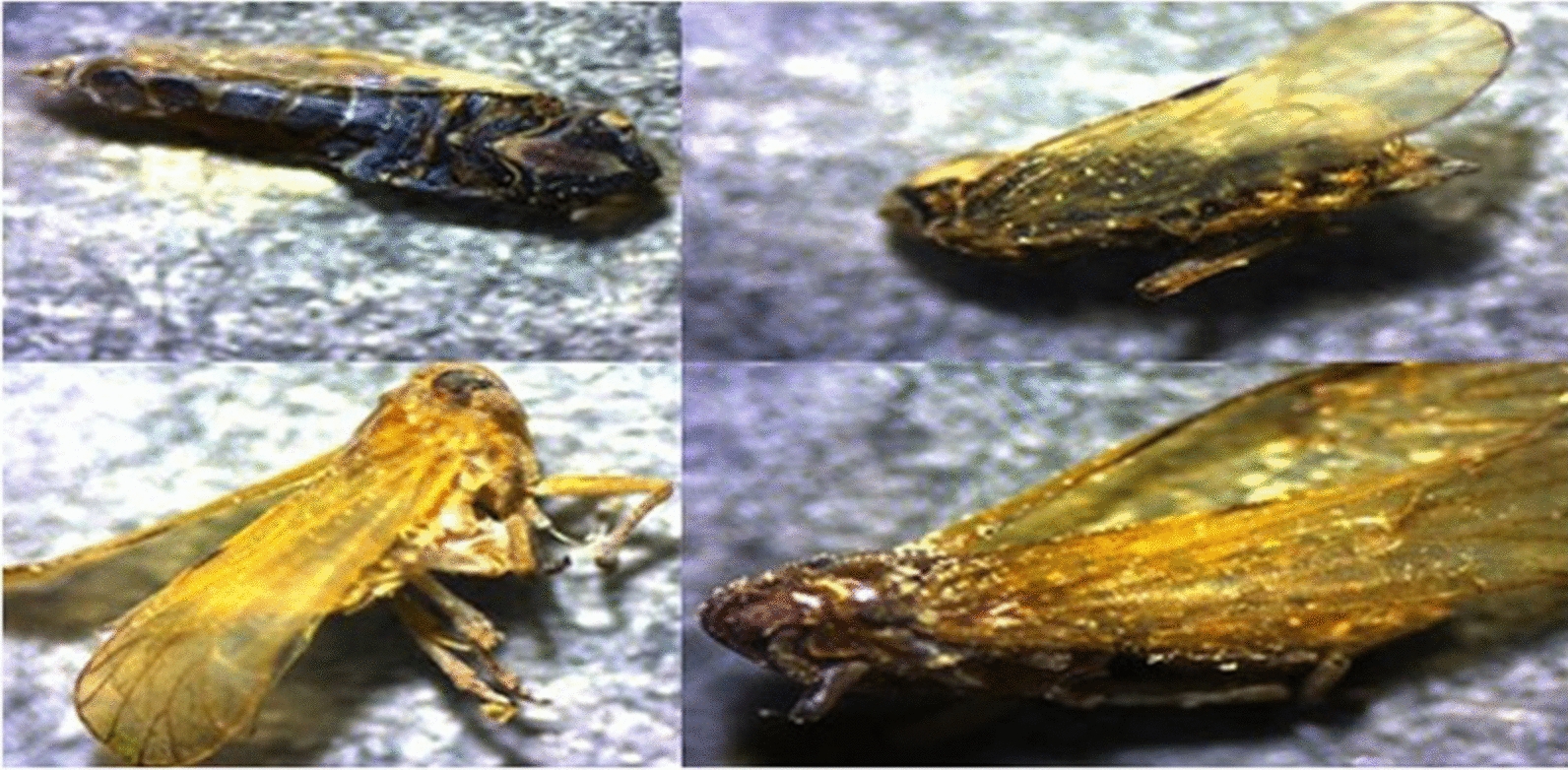
BPH adults dead after treatment of exposure to H_4_L–AgNPs and TBAPy–AgNPs

Immediate toxicity bioassay demonstrated that H_4_L– and TBAPy–AgNPs are toxic to unsexed adult BPH. The concentration of our applied materials and the posttreatment time significantly affected the mortality for all tested adult insects. At high concentrations (1500 and 2000 mg/L), the toxicity of H_4_L (71.25% and 78.50%, respectively) was higher than that of TBAPy (64.50% and 70.50%, respectively) 10 days posttreatment (Additional file 1: Figure S8A). Similarly, at low concentrations (15 and 20 mg/L), H_4_L–AgNPs caused higher mortality (92% and 98%, respectively) compared with TBAPy–AgNPs (70% and 79% mortality, respectively) in BPH adults 10 days posttreatment (Additional file 1Figure S8B). Moreover, TBAPy were less effective against BPH at a high concentration, with an LC_50_ and LC_90_ of 810 and 1982 mg/L, respectively, 168 h posttreatment. However, the toxicity of H_4_L against BPH increased, with an LC_50_ and LC_90_ of 786 and 1919 mg/L, respectively, 168 h posttreatment (Table 1). Furthermore, at a similar concentration of dimethyl sulfoxide as a solvent for the two ligands (negative control), there was no obvious effect on adult BPH 168 h posttreatment. However, H_4_L–AgNPs were highly toxic to BPH, with an LC_50_ and LC_90_ of 3.9 and 14.2 mg/L, respectively, after 168 h posttreatment, while TBAPy–AgNPs were less toxic at the same concentration, with an LC_50_ and LC_90_ of 4.6 and 16.1 mg/L, respectively (Table 2). The regression equation suggested a linear relationship between the rate of mortality and small doses of AgNPs, with a positive relationship between doses, AgNP shapes, and the insect body’s absorption of high quantities of AgNPs that caused their death in the end. Over 15 days of careful observation, 100% mortality was seen in BPH adult at all concentrations of H_4_L– and TBAPy–AgNPs (Fig. 6), indicating that insect mortality might largely be due to the toxic inner effects of the AgNPs inside the cuticle. On the basis of our findings, the performance of AgNPs is strongly correlated with the concentration of AgNO_3_, with the highest activity being achieved with 1 mM for both ligands. In summary, the toxicity and insecticidal activity decreased in the order of 1 mM > 3 mM > 5 mM, which might be due to a decrease in reaction exposure.

**Table 1:** Insecticidal activity of H_4_L and TBAPy solution against brown planthopper *N. lugens* adults

Treatments	Time of exposure	LC_50_ (LC_90_)	95% Confidence limit LC_50_ (LC_90_)				Regression equation		χ^2^ (*df* = 3)		*P*-value		*R*-value
			LCL	UCL									
H_4_L	1 Day	921 (1984)	270 (1307)	1558 (87,741)			Y = -10.8 + 3.8x		7.33		0.03		0.89
	2 Days	886 (1946)	40 (1267)	1695 (92,875)			Y = -10.8 + 3.8x		8.28		0.02		0.94
	5 Days	866 (1942)	38 (1253)	1603 (37,641)			Y = -10.8 + 3.8x		8.06		0.02		0.97
	7 Days	835 (1938)	32 (1218)	1622 (37,617)			Y = -8.4 + 3.0x		8.45		0.02		0.95
	10 Days	786 (1919)	29 (1157)	1425 (77,531)			Y = -8.6 + 3.1x		8.18		0.02		0.98
TBAPy	1 Day	1010 (2351)	360 (1466)	1802 (150,889)			Y = -9.1 + 3.1x		6.75		0.03		0.93
	2 Days	950 (2271)	62 (1361)	1997 (455,175)			Y = -9.1 + 3.1x		8.17		0.02		0.96
	5 Days	892 (2097)	59 (1247)	2006 (505,151)			Y = -9.9 + 3.8x		9.32		0.01		0.97
	7 Days	856 (2073)	54 (1234)	1729 (606,996)			Y = -9.9 + 3.8x		8.66		0.01		0.97
	10 Days	810 (1982)	48 (1193)	1492 (426,414)			Y = -9.9 + 3.8x		8.15		0.02		0.97

LC_50_ lethal concentration that kills 50% of the exposed insects, LC_90_ lethal concentration that kills 90% of the exposed insects, LCL lower confidence limit, UCL upper confidence limit, χ^2^ chi-square test, *df* degrees of freedom, P-value probability value, R-value correlation coefficient

**Table 2:** Insecticidal effects of synthesized H_4_L-AgNPs and TBAPy-AgNPs against brown planthopper *N. lugens* adult

Treatments	Time of exposure	LC_50 (_LC_90_)	95% Confidence limit LC_50_ (LC_90_)		Regression equation	χ^2^ (*df* = 3)	*P*-value	*R*-value
			LCL	UCL				
H_4_L-NPs	1 Day	6.6 (18.3)	5.5 (15.5)	7.6 (23.5)	Y = -2.0 + 2.5x	0.13	0.94	0.99
	2 Days	6.2 (17.8)	4.9 (14.9)	7.2 (23.0)	Y = -2.4 + 3.1x	0.52	0.77	0.99
	5 Days	5.8 (17.8)	4.5 (14.9)	6.8 (23.7)	Y = -1.6 + 2.3x	0.64	0.73	0.98
	7 Days	5.2 (17.0)	3.9 (14.1)	6.3 (22.7)	Y = -1.5 + 2.5x	1.34	0.51	0.97
	10 Days	3.9 (14.2)	2.5 (11.7)	5.1 (19.1)	Y = -1.5 + 2.5x	2.93	0.23	0.99
TBAPy-NPs	1 Day	7.1 (20.0)	5.8 (17.1)	8.1 (27.3)	Y = -2.0 + 2.5x	0.25	0.86	0.96
	2 Days	6.7 (20.0)	5.4 (16.6)	7.7 (26.7)	Y = -2.0 + 2.5x	0.30	0.86	0.98
	5 Days	6.2 (19.9)	4.9 (16.4)	7.3 (27.1)	Y = -2.0 + 2.5x	0.24	0.89	0.97
	7 Days	5.9 (18.5)	4.6 (15.4)	6.9 (24.7)	Y = -2.0 + 2.5x	0.37	0.83	0.97
	10 Days	4.6 (16.1)	3.2 (13.3)	5.7 (21.9)	Y = -1.5 + 2.5x	2.60	0.27	0.99

*LC*_*50*_ lethal concentration that kills 50% of the exposed insects, *LC*_*90*_ lethal concentration that kills 90% of the exposed insects, *LCL* lower confidence limit, *UCL* upper confidence limit, *χ*^*2*^ chi-square test, *df* degrees of freedom, *P-value* probability value, *R-value* correlation coefficient

TEM analysis showed that the agglomeration rate increased with increasing concentration of the Ag precursor (Additional file 1: Figure S4). It is noteworthy that the efficiency of NPs can be negatively affected by such aggregation where specific NPs can participate in the biological reaction, while the remaining become inactive because the reaction takes place on the top layer of the exposed NPs. In contrast, a well-dispersed nanocluster can guarantee full use of the exposed area to the biological process and all the NPs are efficiently involved (Additional file 1: Figure S4). The cross-activity of NPs driving an individual cell can prevent molting and other physiological procedures that accelerate mortality [11, 52]. The LC_50_ was 37.7, 31.3, and 20.7 mg/L after 24 h exposure to AgNPs and 25.5, 21.1, and 7.4 mg/L after 48 h exposure to AgNPs against *Anopheles stephensi*, *Culex quinquefasciatus*, *and Aedes aegypti*, respectively [19]. Similarly, the LC_50_ was 19.7, 24.7, and 26.6 μg/mL and the LC_90_ was 38.8, 53.0, and 60.3 μg/mL against the early second, third, and fourth instars, respectively, in *A. stephensi* [50].

### Inhibitory activity of H_4_L, TBAPy, and synthesized AgNP-based complexes on brown planthopper enzymes

**Fig. 7:**
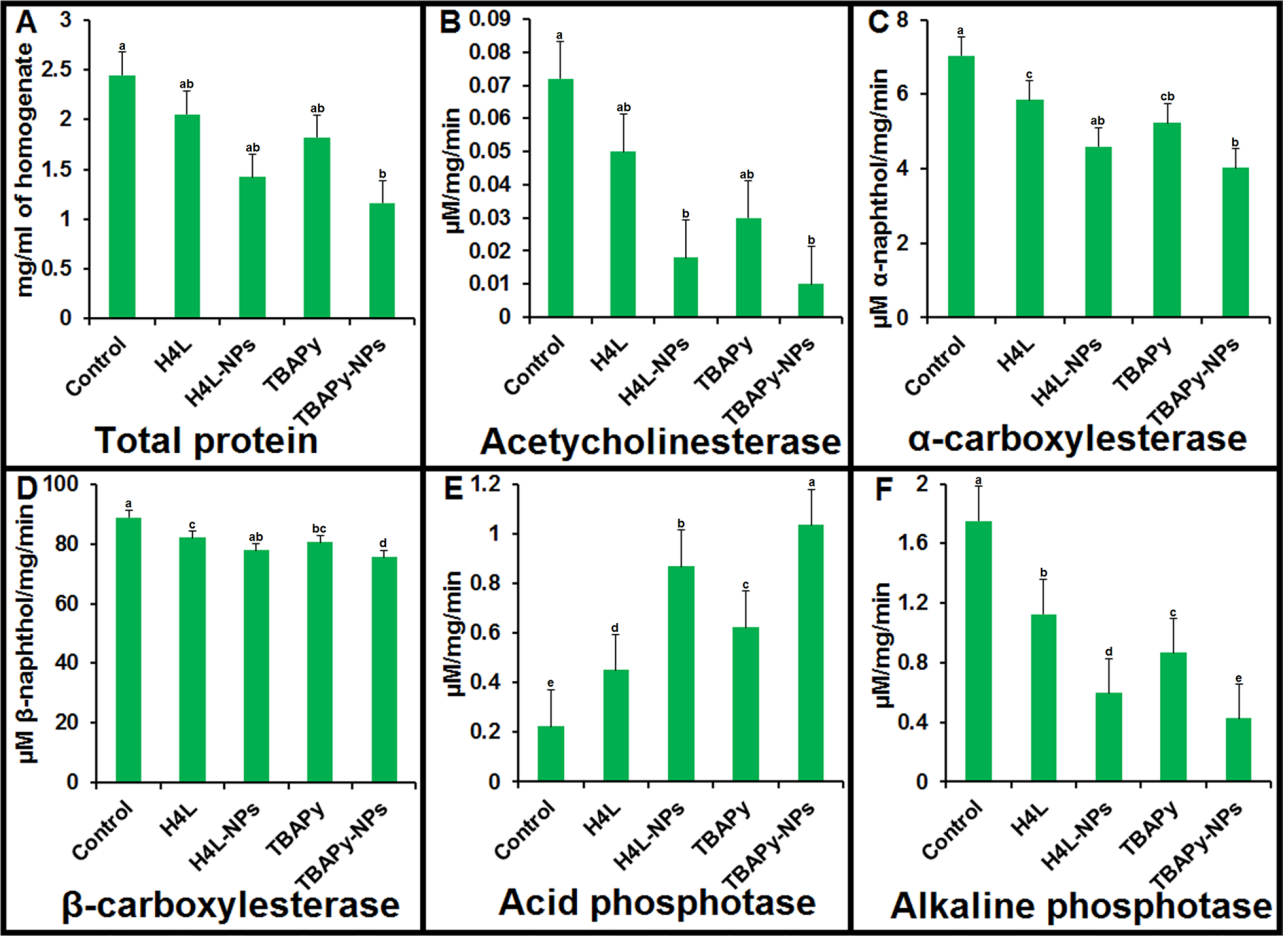
Effect of H_4_L, TBAPy, H_4_L–AgNP, and TBAPy–AgNP on the enzyme activities and total proteins. **A** Total protein concentration, **B** Acetylcholine esterase, **C** α-Carboxylesterase, **D** β-Carboxylesterase, **E** acid phosphatase, and **F** alkaline phosphatase enzyme. Different letters above the bars of each figure indicate significant differences based on Duncan’s test at p ≥ 0.05 between control and other treatments. Each bar represents the mean ± SE of four replicates using different preparations of insect homogenates

Considering the insecticidal activity of H_4_L– and TBAPy–AgNPs, it is also necessary to know their mechanism of action. We measured the effects of AgNPs and ligands on the biochemical constituents of BPH. In general, the tested samples caused variation in the normal biochemical constituents of BPH, with either an increase or a decrease in their activity compared to the controls (Fig. 7). We studied on the effects of H_4_L–AgNPs, TBAPy–AgNPs, and pure H_4_L and TBAPy on different enzymes involved in biological processes in BPH adults and estimated total protein levels, acetylcholinesterase activity, α- and β-carboxylesterase activity, and acid and alkaline phosphatase activity. At 72 h posttreatment, H_4_L– and TBAPy–AgNPs significantly decreased the total protein level from the control value of 2.45 to 1.42 and 1.16 mg protein/mL of homogenate (*p* > 0.05), respectively (Fig. 7A); significantly decreased acetylcholinestrase activity from the control value of 0.072 to 0.018 and 0.01 μM ACT/mg/min (*p* > 0.05), respectively (Fig. 7B); significantly decreased α-carboxylesterase activity from the control value of 7.03 to 4.59 and 4.02 μM α-naphthol/mg/min (*p* > 0.05), respectively (Fig. 7C); and slightly decreased β-carboxylesterase activity from the control value of 89.02 to 77.86 and 75.55 µM β-naphthol/mg/min (*p* > 0.05), respectively (Fig. 7D). Esterases are the primary enzymes involved in the development of resistance mechanisms to chemical insecticides by splitting carboxyl ester and phosphodiester bonds [53]. We found that H_4_L– and TBAPy–AgNPs are able to highly reduce α- and β-carboxylesterase activity, and the level of detoxifying enzymes is significantly down regulated during BPH development. Hassan et al. treated mosquitoes with salicylic acid (SA), 3,5-dinitrosalicylic acid (DNS), and AgNPs and found reduced levels of acetylcholinesterase after treatment with SA– and DNS–AgNPs compared with the controls. They also found that α- and β-carboxylesterase activity was highly inhibited after treatment with SA– and DNS–AgNPs to 0.33 and 0.30 µM α,β-naphthol/min/mg protein, respectively, compared with the controls [54].

At 72 h posttreatment, H_4_L– and TBAPy–AgNPs significantly increased acid phosphatase activity from the control value of 0.224 to 0.869 and 1.035 mM/mg/min (*p* > 0.05), respectively (Fig. 7E), while they decreased alkaline phosphatase activity from the control value of 1.751 to 0.596 and 0.426 mM/mg/min (*p* > 0.05), respectively (Fig. 7F). Acid and alkaline phosphatases play an important role in the hydrolytic cleavage of phosphoric acid esters and regulate the acid–alkali balance [55]. In addition, these enzymes are important for many significant physiological processes, such as metabolism and cellular signaling [56].

### AgNP accumulation in brown planthopper by TEM

**Fig. 8:**
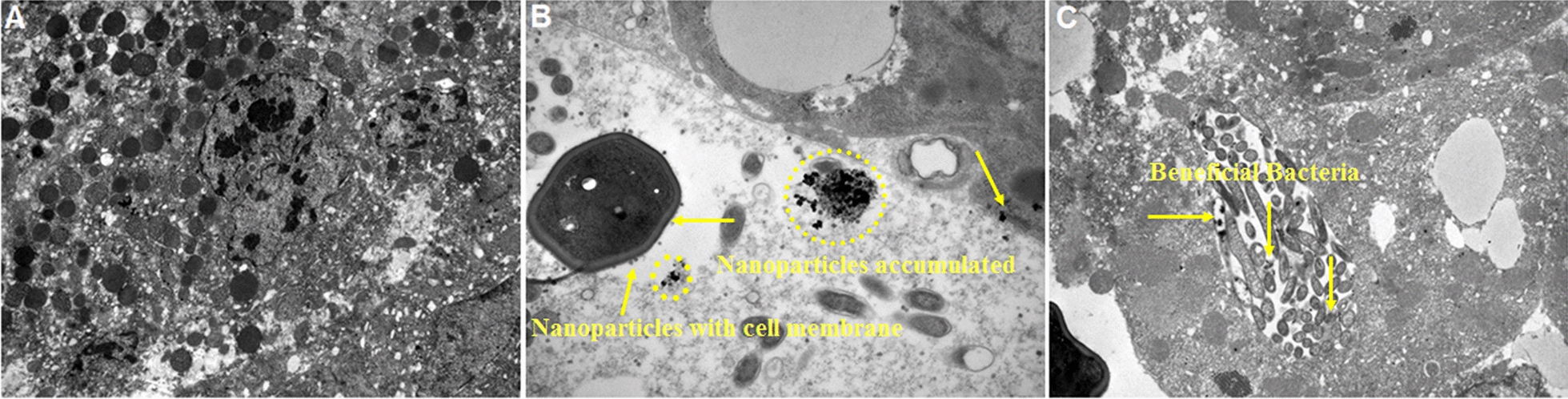
TEM images of H_4_L–AgNP, TBAPy–AgNP treated adult, and untreated insect (control). **A** Ultrastructure of the midgut in control showing midgut microvilli and different cell organelles with normal characteristics. **B**, **C** Accumulation of AgNPs in the endoplasmic reticulum, nucleolus, and other cell organelles of gut cells pointed by yellow arrows and circles (dark spots)

The LC_30_ exposure and control of BPH were used for TEM analysis to observe AgNP accumulation in the midgut cells of BPH adults. Figure 8A shows the microvilli structures in the midgut region of the control group. H_4_L– and TBAPy–AgNP-treated BPH showed accumulation of Ag particles in the midgut cells as dark spots, indicated by yellow arrows and circles, respectively. We observed distinct localization and accumulation of AgNPs in the rough endoplasmic reticulum (ER), in addition to beneficial bacteria (Figs. 8B, C). Our results are in agreement with the findings of Yasur et al. [36], who observed the accumulation of AgNPs in the rough ER and some other cell organelles of the midgut cells of AgNP-treated *Achaea janata* larvae. Similarly, gold (Au) NPs accumulated in the rough ER and cell vesicles of AuNP-treated *Drosophila melanogaster* [57]. TEM insect images showed that the AgNPs absorbed by midgut cells localize and accumulate in the cells’ organelles, which could lead to detrimental effects, including DNA damage, genotoxicity, mitochondrial dysfunction, altered cell morphology, and subsequent necrosis or apoptosis [58].

### Histological studies

**Fig. 9:**
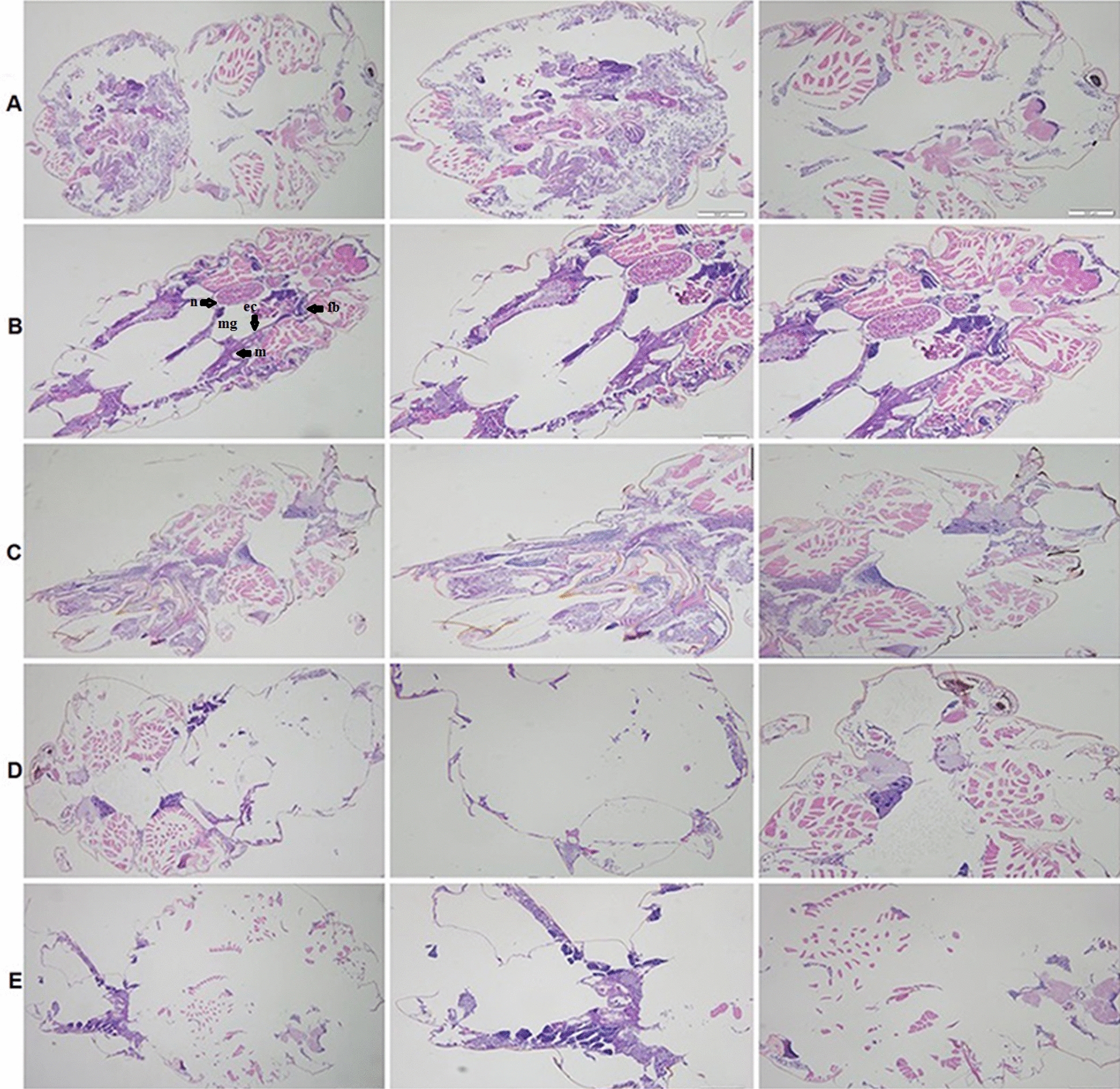
Longitudinal sections of the midgut of brown planthopper *N. lugens*, magnification at 40x. **A** Control and **B**−**E** H_4_L, TBAPy, H_4_L–AgNP, and TBAPy–AgNP treatments. Graph showing the treated insects by two kinds of nanoparticles which highly affected the midgut, epithelial cells, and it showed cytopathological variations, and degradation of nuclei. Midgut (mg); fat body (fb); epithelial cells (ec); muscles (m); nucleus (n)

The LC_50_ exposure and control of BPH for 72 h were used for histological analysis. We found histological structure alterations in the midgut epithelial cells in treated insects (Fig. 9). The midgut area, including epithelial cells and the brush border, were significantly affected by H_4_L– and TBAPy–AgNPs compared with the controls. The midgut epithelium of the controls exhibited normal cytoplasmic characteristics with a regular microvilli lining (Fig. 9A) at 40 × magnification. H_4_L, TBAPy, H_4_L–AgNPs, and TBAPy–AgNPs caused histological alterations in the midgut, such as elongation of epithelial cells protruding into the lumen and disintegration of the brush border (Figs. 9B, C). TBAPy–AgNPs partially destroyed the epithelial cells in the posterior region of the midgut (Fig. 9D). Notably, H_4_L–AgNPs caused severe damage in the midgut epithelial cells, with cytopathological variations, such as destruction of epithelial cells and degradation of nuclei (Fig. 9E). Severe lesions were also seen in the midgut epithelial cells, including broken membranes, brush border damage, and vacuolization (Fig. 9D, E).

## Conclusions

This paper highlights AgNP synthesis via a liquid-phase reduction approach using two new alternative organic ligands H_4_L and TBAPy as reducing agents, obtained by different chemical techniques. These two ligands can directly transform dissolved Ag ions into quasi-spherical architectures. H_4_L– and TBAPy–AgNPs were used as insecticides to control the BPH population. At the same concentration, H_4_L–AgNPs are more toxic than TBAPy–AgNPs after 10 days of direct contact with BPH. Furthermore, the toxicity increases with increasing doses. In addition, H_4_L– and TBAPy–AgNPs significantly affect the midgut of BPH compared with H_4_L and TBAPy alone and cause cytopathological variations, such as destruction of epithelial cells and degradation of nuclei. Our findings provide insights into the mechanism of action of AgNPs, which operate by affecting enzymatic pathways. AgNP–ligand formulations produce a synergetic effect to combat the adverse effects of bulk insecticide on the environment. AgNP-intercalated organic ligands present a new and potential tool for protecting rice plants from pests and may be used as an eco-friendly insecticide at low doses to reduce the BPH population in rice fields.

## Supplementary Information

**Additional file 1. Scheme S1.** [I] DMF, sodium carbonate, palladium acetate, 60 °C; [II] Extracted with ethyl acetate, Synthesis of 2’-Amino-1,1’:4’,1’’-terphenyl-3,3’’,5,5’’-tetracarboxylic acid (H_4_L). **Scheme S2.** Bromination and synthesis of 1,3,6,8-tetrakis (*p*-benzoic acid)pyrene (TBAPy). **Figure S1. **^1^H NMR, for the 2’-amino-1,1’:4’,1’’-terphenyl-3,3’’,5,5’’-tetracarboxylate.** Figure S2.** ^1^H NMR, for the 1,3,6,8-tetrakis (4-(methoxycarbonyl)phenyl)-pyrene. **Figure S3.** ^1^H NMR, for the 1,3,6,8-tetrakis (*p*-benzoic acid)-pyrene. **Figure S4.** TEM images of the as-prepared H_4_L–AgNPs complexes with different AgNO_3_ concentrations. (**A**) 1 mmol, (**B**) 3 mmol, and (**D**) 5 mmol. **Figure S5. (A, B)** SEM image and elemental mappings of the as-obtained H_4_L–AgNPs illustrating the composition of the final product and the corresponding distribution of the observed Ag (**C)**, Al (**D**), Cl (**E**), S (**F)**, and O (**G)** components. (**H)** Plots of intensity versus the cross-sectional compositional line of the complex. **Figure S6.** EDX patterns of synthesized silver nanoparticles (**A)** H_4_L–AgNPs and (**B)** TBAPy–AgNPs. **Figure S7. **Physico‑chemical characterization of the nanoparticles. A and B: DLS profiles of the size distribution of H_4_L–AgNP and TBAPy–AgNP. C and D: Stability evaluation of Zeta potential analysis of H_4_L–AgNP and TBAPy–AgNP. **Figure S8.** Graph showing the mortality % of brown planthopper adults** (A)** H_4_L and TBAPy solution at 500, 1000, 1500, 2000 mg/L; (**B)** silver nanoparticles at 5, 10, 15, 20 mg/L; The mortality data for DMSO or control treatments were less than 10%. Different letters above the bars of each figure indicate significant differences based on Duncan’s test at p≥ 0.05 between concentrations (mg/L) and different days. Each bar represents the mean ±SE of four replicates.

## Data Availability

The datasets used and/or analysed during the current study are available from the corresponding author on reasonable request.

## Abbreviations

^1^H NMR: Proton nuclear magnetic resonance
AgNO_3_: Silver nitrate
AgNP: Silver nanoparticle
ANOVA: Analysis of variance
BPH: Brown planthopper
DMF: Dimethylformamide
DTNB: 5,5′-Dithiobis (2-nitrobenzoic acid)
EDX: Energy-dispersive X-ray spectroscopy
FT-IR: Fourier transform infrared spectroscopy
H_4_L: 2’-Amino-1,1’:4’,1’’-terphenyl-3,3’’,5,5’’-tetracarboxylic acid
LC_50_: Median lethal concentration
L:D: Light:day
NP: Nanoparticle
PBS: Phosphate-buffered saline
PMOF: Porous metal–organic framework
ppm: Parts per million
SBU: Secondary bonding unit
SEM: Scanning electron microscopy
SPR: Surface plasmon resonance
TBAPy: 1,3,6,8-Tetrakis (*p*-benzoic acid)-pyrene
TEM: Transmission electron microscopy
TMS: Trimethylsilyl chloride
XRD: X-ray diffraction
