# Supplementary material for: Green synthesis of AgNP–ligand complexes and their toxicological effects on Nilaparvata lugens
Source: J Nanobiotechnology. 2021 Oct 13;19:318. doi: 10.1186/s12951-021-01068-z (PMC8513204; doi:10.1186/s12951-021-01068-z)

**Supplementary Materials.**

**Green synthesis of AgNP–ligand complexes and their toxicological effects on *Nilaparvata lugens***

**Hatem Fouad^1,2*^, Guiying Yang^1^, Ahmed A. El-Sayed^3^, Guofeng Mao^1^, Diab Khalafallah^4^, Mahmoud Saad^2^, Hassan Ga’al^1^, Ezzeldin Ibrahim^5^, Jianchu Mo^1^***

^1^Ministry of Agriculture, Key Laboratory of Molecular Biology of Crop Pathogens and Insect Pests, Institute of Insect Sciences, College of Agriculture and Biotechnology, Zhejiang University, Hangzhou, Zhejiang, China

^2^Department of Field Crop Pests, Plant Protection Research Institute, Agricultural Research Centre 12622, Dokki, Cairo, Egypt

^3^Photochemistry Department, National Research Center, Dokki, Giza, Egypt

^4^State Key Laboratory of Silicon Material, School of Materials Science and Engineering, Zhejiang University, Hangzhou, China

^5^State Key Laboratory of Rice Biology and Ministry of Agriculture Key Lab of Molecular Biology of Crop Pathogens and Insects, Institute of Biotechnology, Zhejiang University, Hangzhou 310058, China

Corresponding author

*Jianchu Mo

Ministry of Agriculture Key Laboratory of Molecular Biology of Crop Pathogens and Insect Pests, Institute of Insect Sciences, College of Agriculture and Biotechnology, Zhejiang University, Yuhangtang Road 866, Hangzhou, Zhejiang 310058, P. R. China; Tel/Fax: +86-571-88982695; E-mail: [mojianchu@zju.edu.cn](mailto:mojianchu@zju.edu.cn)

*Hatem Fouad

Ministry of Agriculture Key Laboratory of Molecular Biology of Crop Pathogens and Insect Pests, Institute of Insect Sciences, College of Agriculture and Biotechnology, Zhejiang University, Yuhangtang Road 866, Hangzhou, Zhejiang 310058, P. R. China; Tel/Fax: +86-571-88982695; Email: [dr_hatem@zju.edu.cn](mailto:dr_hatem@zju.edu.cn)

**Abstract**

**Background:** Despite developments in nanotechnology for use in the pharmaceutical field, there is still a need for implementation of this technology in agrochemistry. In this study, silver nanoparticles (AgNPs) were successfully prepared by a facile and an eco-friendly route using two different ligands, 2’-amino-1,1’:4’,1’’-terphenyl-3,3’’,5,5’’-tetracarboxylic acid (H_4_L) and 1,3,6,8-tetrakis(*p*-benzoic acid)-pyrene (TBAPy), as reducing agents. The physiochemical properties of the as-obtained AgNPs were characterized by scanning electron microscopy (SEM), energy-dispersive X-ray (EDX), X-ray diffraction (XRD) and transmission electron microscopy (TEM). The toxicity of H_4_L–AgNP and TBAPy–AgNP against the brown planthopper (BPH, *Nilaparvata lugens*) was also measured.

**Results:** SEM and TEM analyses demonstrated the formation of quasi-spherical AgNP structures in the presence of H_4_L and TBAPy. Insecticidal assays showed that TBAPy is less effective against *N. lugens*, with a median lethal concentration (LC_50_) of 810 mg/L, while the toxicity of H_4_L increased and their LC_50_ reached 786 mg/L 168 h posttreatment at a high concentration of 2000 mg/L. H_4_L–AgNPs were also highly toxic at a low concentration of 20 mg/L, with LC_50_ = ~3.9 mg/L 168 h posttreatment, while TBAPy–AgNPs exhibited less toxicity at the same concentration, with LC_50_ = ~4.6 mg/L.

**Conclusions:** These results suggest that the synthesized AgNPs using the two ligands may be a safe and cheaper method compared with chemical insecticides for protection of rice plants from pests and has potential as an effective insecticide in the *N. lugens* pest management program.

**Keywords:** Nanotechnology; Organic ligands; Silver nanoparticles; Insecticidal effect; *Nilaparvata lugens*

**Materials and Methods**

**Synthesis of 2’-Amino-1,1’:4’,1’’-terphenyl-3,3’’,5,5’’-tetracarboxylic acid (H_4_L)**

1. **3,5-bis(ethoxycarbonyl)-phenylboronic acid**: 3,5-dimethylboronic acid (17 mmol) and sodium hydroxide (68 mmol) were dissolved in 250 mL tertbutanol/water (v/v= 1:1) under magnetic stirring. The reaction mixture was heated up to 50 °C until the color changed from brown to red, and then 1 g of KMnO_4_ was added to the mixture. After that, 25 g of KMnO_4_ was introduced, and the temperature was raised to 70 °C before immersing an additional 3.2 g KMnO_4_ to the reaction. The reaction continued till the brown color persisted for 3 h. Subsequently, the hot solution was filtered, and the resulting filtrate was concentrated to 50 mL in a vacuum and acidified by concentrated HCl until the pH of the reaction reached 1. The resulting white precipitate was centrifuged and washed with deionized water before impregnating in a mixture of 100 mL ethanol and 3 mL concentrated H_2_SO_4_ for 12 h with reflux. After cooling down to room temperature, the volume was reduced till 50 mL and then 100 mL deionized water was added to obtain the final product 3,5-bis (ethoxycarbonyl)-phenylboronic acid as confirmed by ^1^H-NMR， (CDCl_3_-d): δ 3.92 (s, 2H), 7.98 (s, 3H), 10.84 (s, 2H). ^13^C-NMR， (CDCl_3_-d): 128.6, 132.2, 133.9, 149.1, 171.3.
2. **2’-amino-1,1’:4’,1’’-terphenyl-3,3’’,5,5’’-tetracarboxy-late:** 3,5-bis(ethoxycarbonyl)-phenylboronic acid (11.95 mmol) and 2,5-dibromoaniline (3.38 mmol) were dissolved in 40 mL dimethylformamide (DMF) and then mixed with another solution containing sodium carbonate (15.94 mmol) and palladium acetate (30 mg) in 60 mL deionized water. The whole mixture was stirred overnight at 60 °C before cooling down to room temperature. Thereafter, 150 mL of deionized water was added to the mixture, and the aqueous phase was extracted three times with ethyl acetate. The organic phase was evaporated to obtain the crude product purified by column chromatography using silica gel (200 mesh). Elution with 30% ethyl acetate in n-hexane was carried out to attain 85% yield of ester as confirmed by ^1^H NMR (DMSO-d_6_): δ 1.25-1.45 (m, 12H), 4.38-4.47 (m, 8H), 5.58 (d, 2H), 7.02 (d, 1H), 7.20 (d, 1H), 7.25 (s, 1H), 8.37 (s, 1H), 7.21 (d, 1H), 7.32 (d, 1H), 7.73 (s, 1H), 8.24 (d, 1H), 8.55 (m, 1H). ^13^C-NMR， (DMSO-d_6_): δ 14.1, 61.4, 106.0, 107.8, 113.1, 115.6, 117.0, 119.3, 120.9, 130.8, 132.9, 138.0, 140.8, 146.4, 147.4, 164.5.
3. **2’-Amino-1,1’:4’,1’’-terphenyl-3,3’’,5,5’’-tetracarboxylic acid, (H_4_L)** Tetra-ester was suspended in a mixture of methanol and deionized water followed by addition of KOH and the reaction mixture was refluxed overnight. After removing the solvent under vacuum, deionized water was added to dissolve the entire solid and subsequently acidified till the final pH became 1. The obtained light-yellow precipitate, namely H_4_L, was collected, filtered, and repeatedly washed with deionized water as well as absolute ethanol as confirmed by ^1^H NMR (DMSO-d_6_): δ 5.62 (d, 2H), 6.60 (d, 1H), 6.63 (d, 1H), 6.92 (s, 1H), 7.05 (s, 1H), 7.30 (d, 1H), 7.42 (d, 1H), 7.70 (s, 1H), 8.44 (d, 1H), 8.65 (m, 1H), 11.83 (s, 4H). ^13^C-NMR， (DMSO-d_6_): δ 106.9, 108.8, 112.1, 114.9, 116.7, 119.9, 121.1, 130.2, 132.6, 137.8, 139.8, 145.2, 146.4, 165.2.

**Synthesis of 1,3,6,8-tetrakis(*p*-benzoic acid)pyrene (TBAPy)**

1. **1,3,6,8-tetrabromopyrene**: Pyrene (25 mmol) and nitrobenzene 175 mL were mixed in a three-necked flask equipped with an external stirrer. 50 mL bromine solution in nitrobenzene (22.5 mmol) was added drop-wise to the above Pyrene solution at 80 °C using a funnel. The formed yellow suspension was heated at 120 °C for 16 h and then cooled down to room temperature. The resulting suspension was separated, and the obtained solid yield was rinsed thoroughly with ethanol and dried under vacuum to get 1,3,6,8-tetrabromopyrene 90% yield as confirmed by ^1^H-NMR， (CDCl_3_-d): δ 6.80 (d, 2H), 7.79 (d, 2H), 8.21 (d, 2H), 8.45 (d, 2H). ^13^C-NMR， (CDCl_3_-d): δ 36.5, 122.6, 124.5, 125.6, 129.8, 132.2, 134.7.
2. **1,3,6,8-tetrakis(4-(methoxycarbonyl)phenyl)-pyrene**: A mixture of 4-methoxycaronylphenyl boronic acid (5.8 mmol), 1,3,6,8-tetrabromopyrene (0.97 mmol), tetrakis (triphenylphosphine) palladium, (0.026 mmol), and potassium tribasic phosphate anhydrous (5.3 mmol) in dry 1,4-dioxane (20 mL) was loaded into 20 mL Teflon-lined stainless steel autoclave and heated under autogenously pressure to 130 °C for 72 h. The reaction mixture was evaporated to dryness, and the solid residue was washed with water to remove the inorganic salts. The insoluble material was extracted with 50 mL chloroform three times, and the organic layer was dried over magnesium sulfate anhydrous before being evaporated under vacuum. Then, the harvested residue was boiled in tetrahydrofuran (THF) for 2 h and filtered to achieve 1,3,6,8-tetrakis (4-(methoxycarbonyl)phenyl) Pyrene (yield 85%) as confirmed by ^1^H-NMR， (CDCl_3_-d): δ 3.99 (s, 12H), 7.77 (d, 8H), 8.01 (s, 2H), 8.15 (s, 4H), 8.23 (d, 8H). ^13^C-NMR， (CDCl_3_-d): δ 10.2, 39.5, 62.3, 113.6, 121.9, 122.6, 124.5, 126.8, 127.9, 128.9, 129.6, 129.8, 130.4, 131.3, 131.2, 133.4, 136.7, 144.3, 149.5, 170.6.
3. **1,3,6,8-tetrakis(*p*-benzoic acid)-pyrene**: To a 250 mL round bottom flask containing 1,3,6,8-tetrakis(4-(methoxycarbonyl)phenyl) Pyrene (0.78 mmol), a solution containing NaOH (37.5 mmol) in 100 mL of THF/water (v/v=1:1) was added and the resultant suspension was vigorously stirred overnight. The solvents were removed under vacuum, deionized water was added to the residue, and a clear yellow solution was formed. The clear yellow solution was stirred at room temperature for 2 h, and the pH value was adjusted to 1 using a concentrated HCl. The resulting yellow solid was collected by ﬁltration and rinsed with deionized water several times. The crude product was recrystallized from DMF, filtered, washed with chloroform, and finally dried under vacuum. This reaction guaranteed 0.49 g (91%) of the pure product 1,3,6,8-tetrakis(*p*-benzoic acid)Pyrene as confirmed by ^1^H NMR (DMSO-d_6_): δ 7.86 (d, 8H), 8.09 (s, 2H), 8.17 (d, 8H), 8.21 (s, 4H), 13.12 (s, 4H). ^13^C-NMR， (DMSO-d6): δ 40.6, 114.3, 120.5, 122.3, 124.9, 127.8, 128.6, 128.9, 129.2, 129.8, 131.1, 131.5, 131.8, 133.7, 136.4, 145.4, 149.1, 171.9.

**Results and discussion**

**Synthesis of H_4_L and TBAPy**

**Scheme S1.** [I] DMF, sodium carbonate, palladium acetate, 60 °C; [II] Extracted with ethyl acetate, Synthesis of 2’-Amino-1,1’:4’,1’’-terphenyl-3,3’’,5,5’’-tetracarboxylic acid (H_4_L).

**
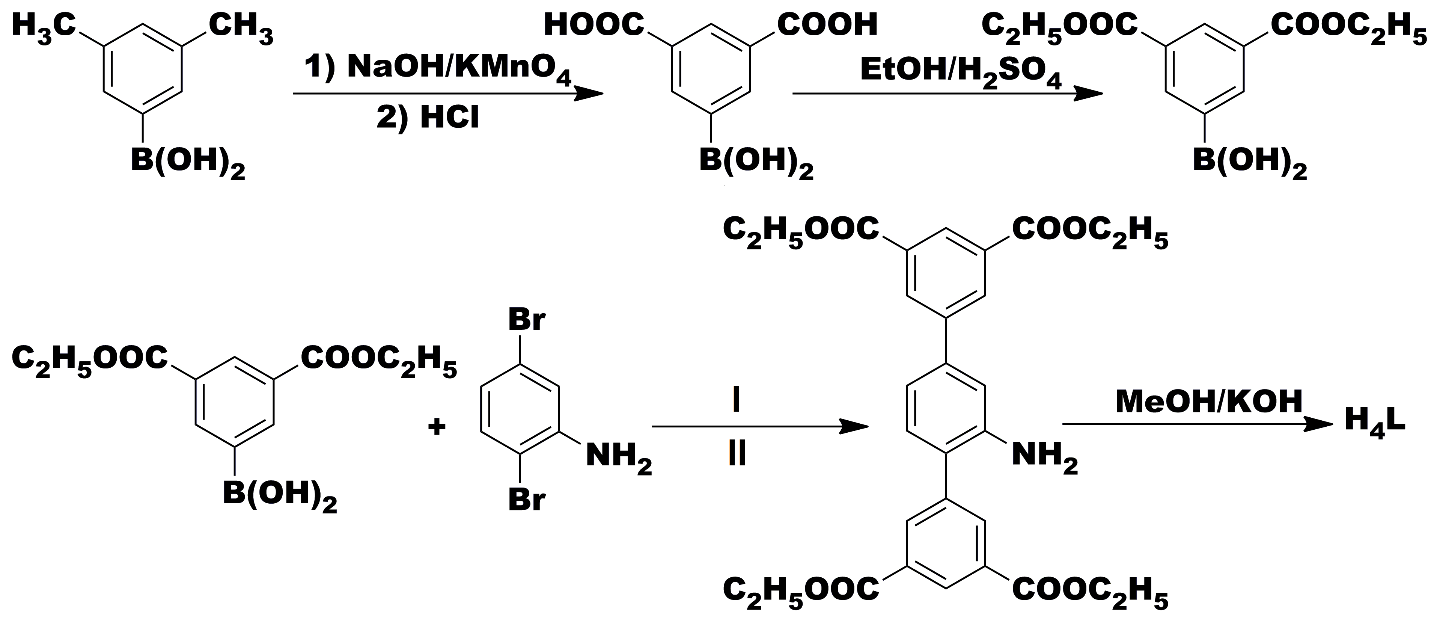
**

**
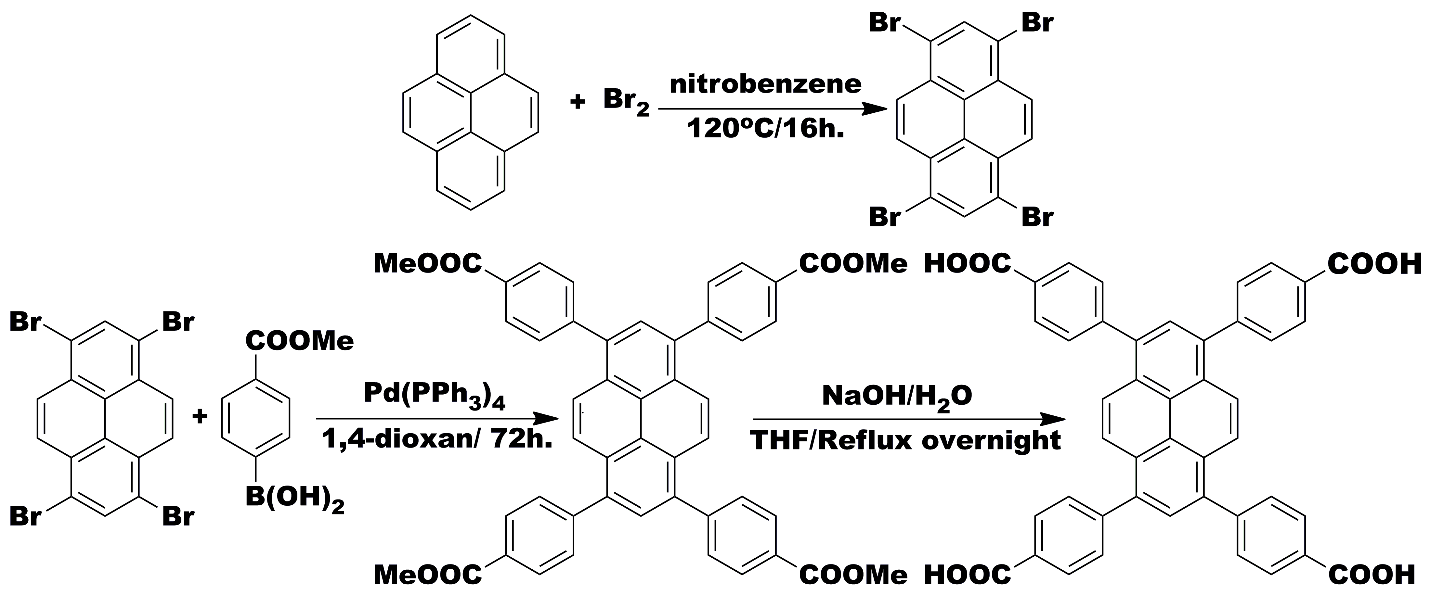
Scheme S2.** Bromination and synthesis of 1,3,6,8-tetrakis(*p*-benzoic acid)pyrene (TBAPy).

**Figure S1.** ^1^H NMR, for the 2’-amino-1,1’:4’,1’’-terphenyl-3,3’’,5,5’’-tetracarboxylate


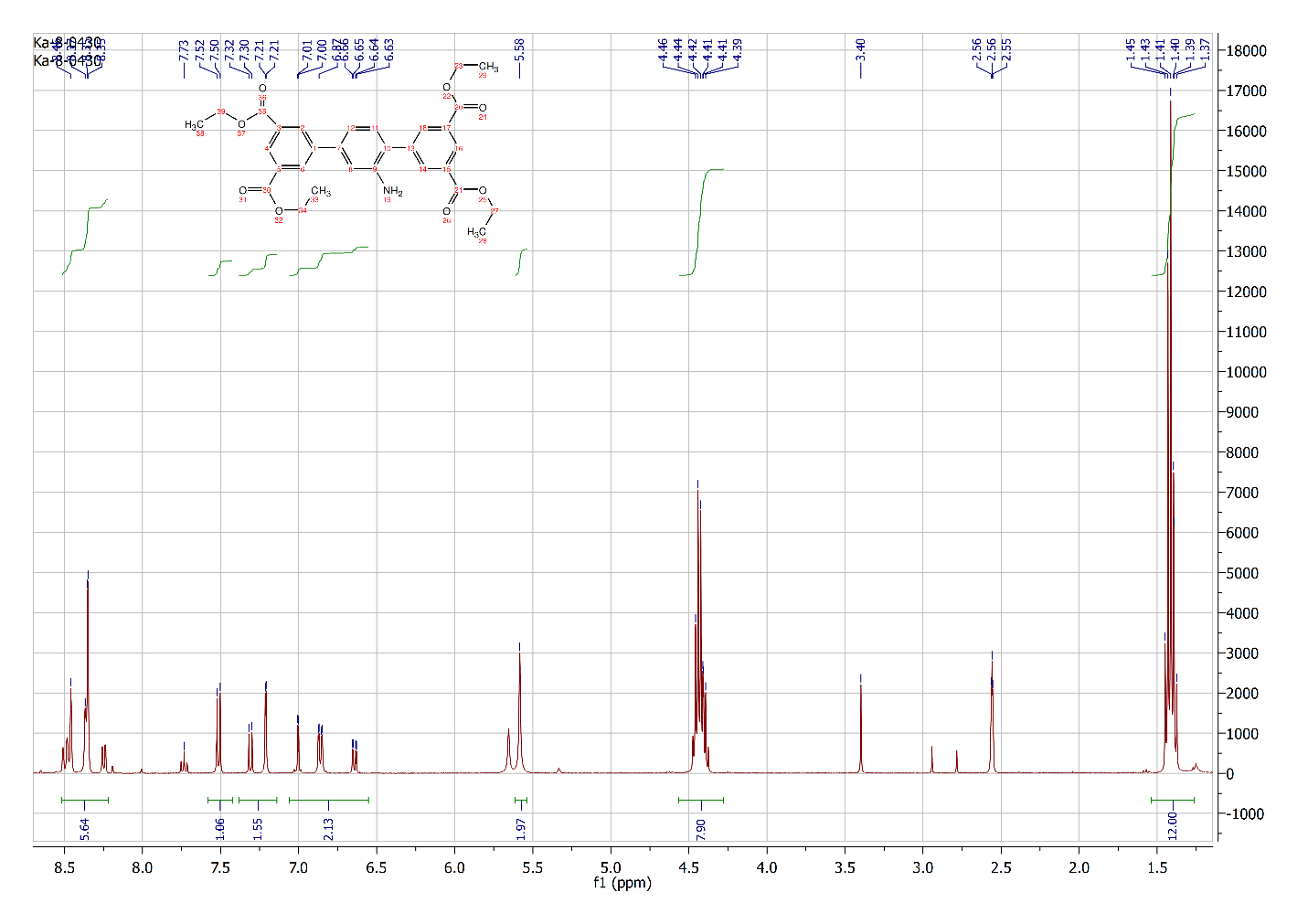


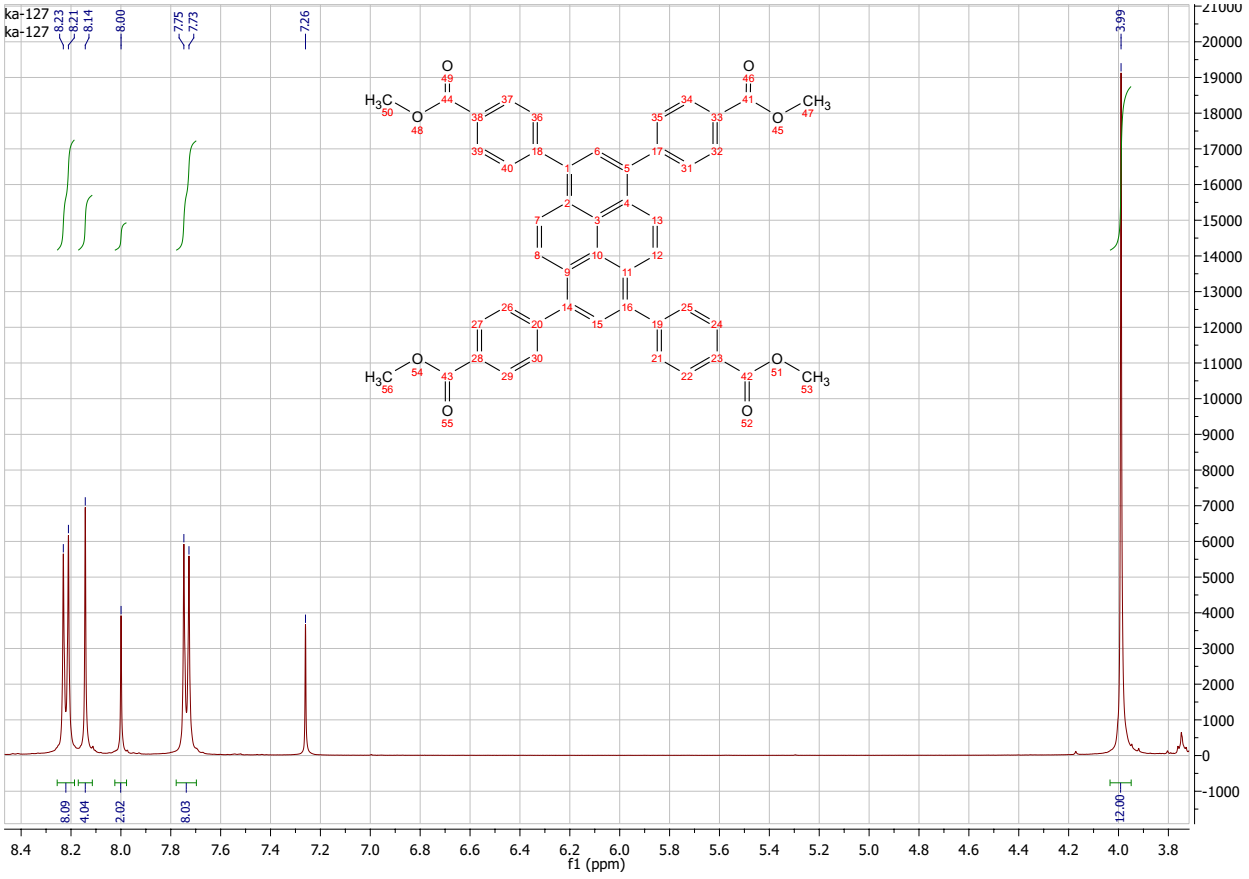
**Figure S2.** ^1^H NMR, for the 1,3,6,8-tetrakis(4-(methoxycarbonyl)phenyl)-pyrene.


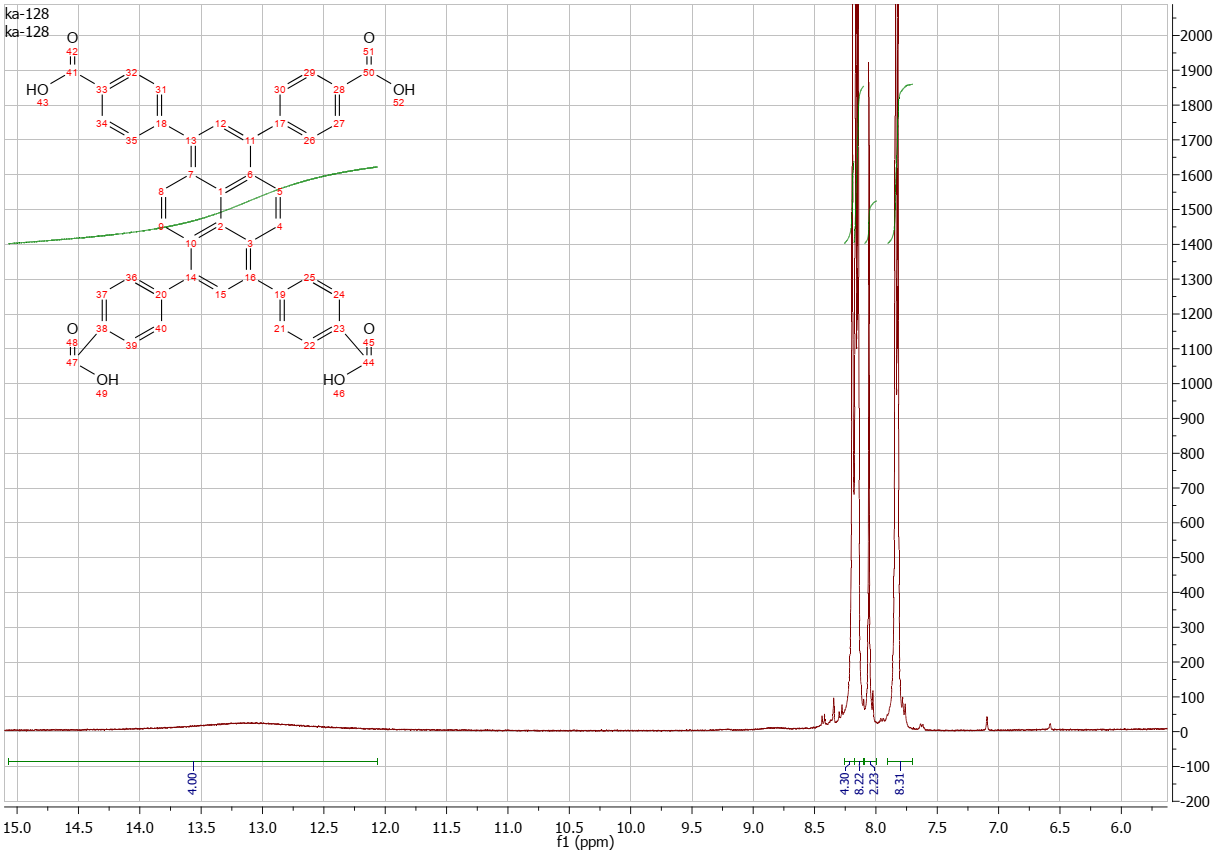
**Figure S3.** ^1^H NMR, for the 1,3,6,8-tetrakis(*p*-benzoic acid)-pyrene.

**Figure S4.** TEM images of the as-prepared H_4_L–AgNPs complexes with different AgNO_3_ concentrations. (**A**) 1 mmol, (**B**) 3 mmol, and (**D**) 5 mmol.


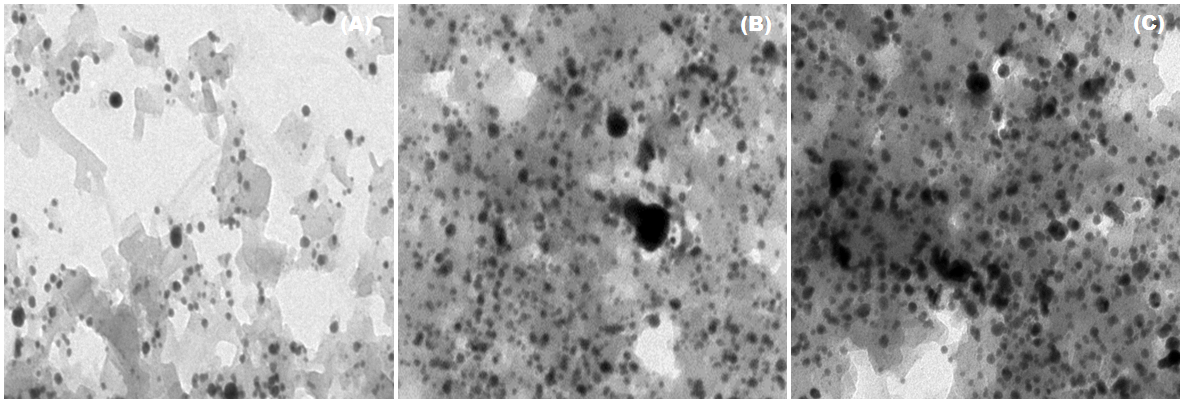


**Figure S5. (A, B)** SEM image and elemental mappings of the as-obtained H_4_L–AgNPs illustrating the composition of the final product and the corresponding distribution of the observed Ag **(C)**, Al **(D)**, Cl **(E)**, S **(F)**, and O **(G)** components. **(H)** Plots of intensity versus the cross-sectional compositional line of the complex.


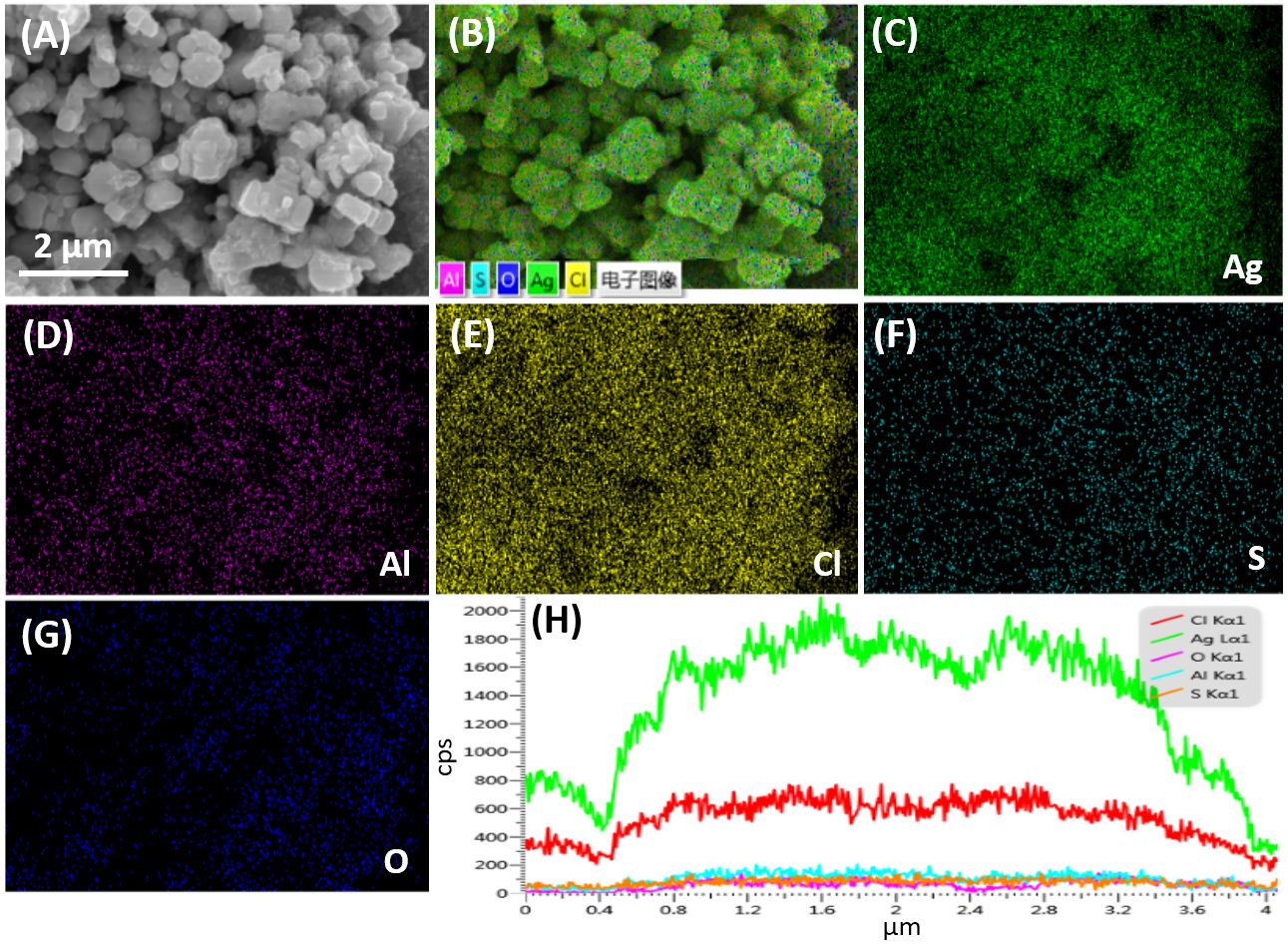


**Figure S6.** EDX patterns of synthesized silver nanoparticles **(A)** H_4_L–AgNPs and **(B)** TBAPy–AgNPs.


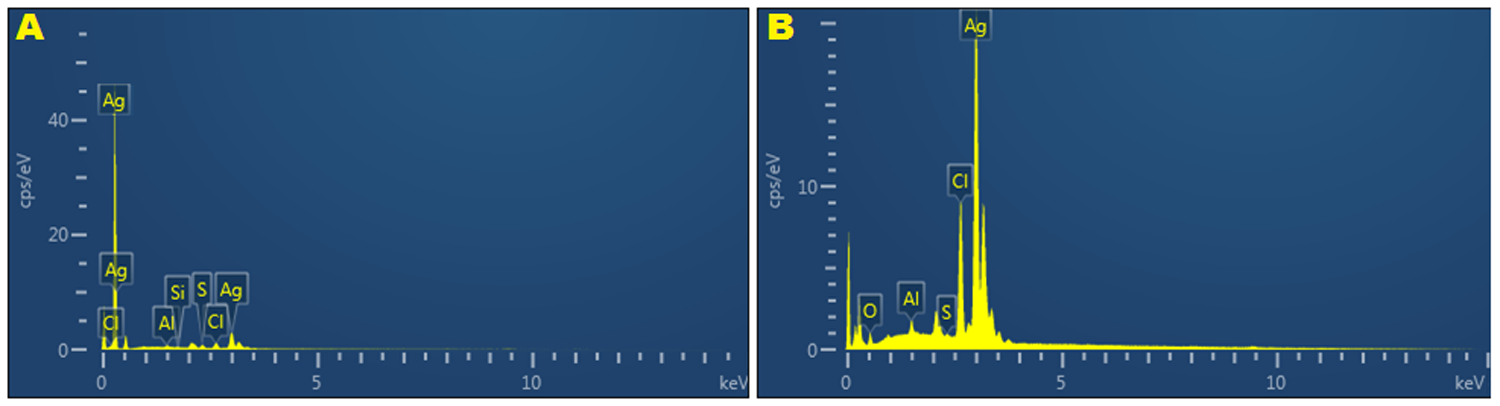


**Figure S7.** Physico‑chemical characterization of the nanoparticles. a and b: DLS profiles of the size distribution of H_4_L–AgNP and TBAPy–AgNP. c and d: Stability evaluation of Zeta potential analysis of H_4_L–AgNP and TBAPy–AgNP.

**
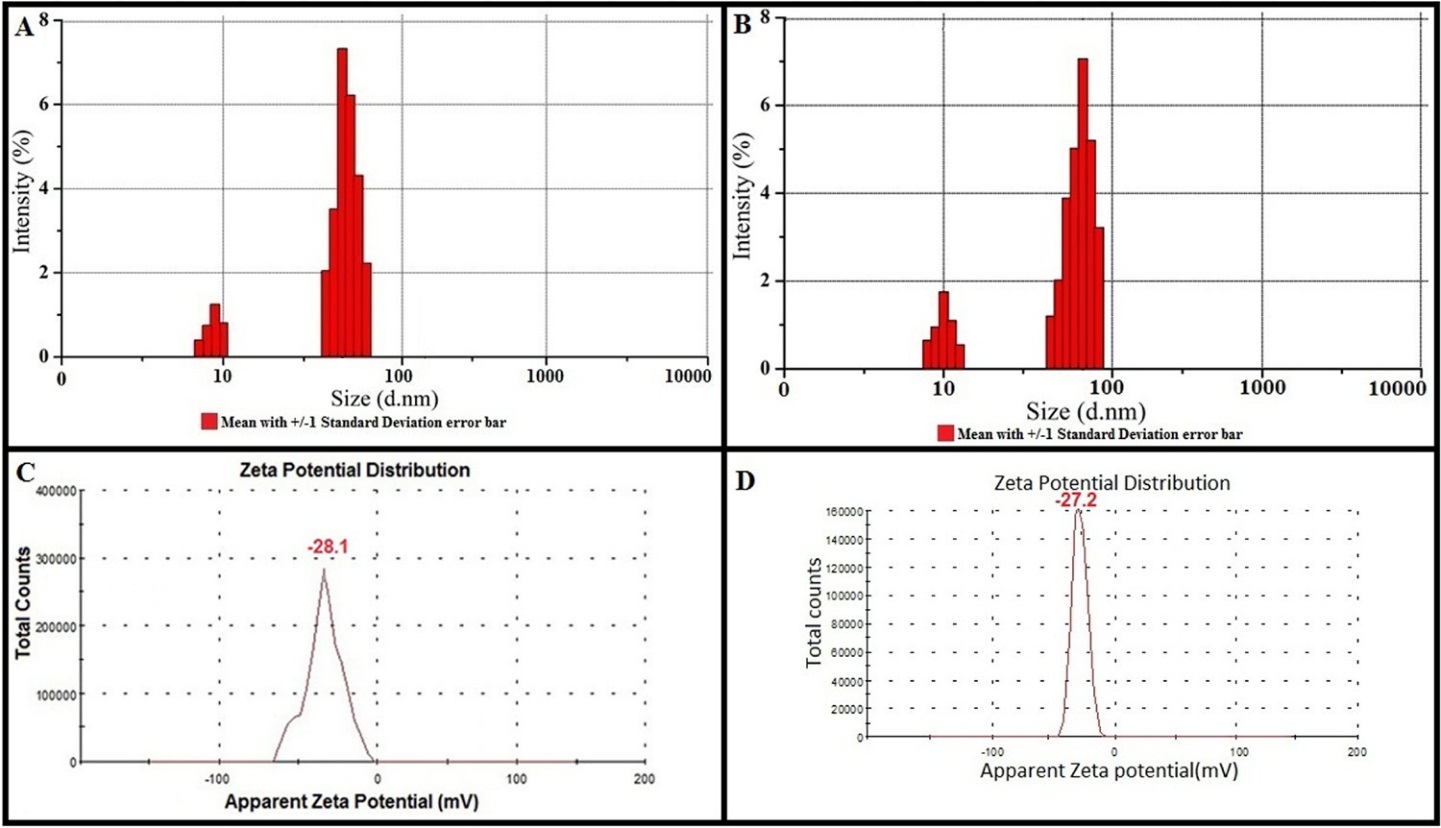
**

**Figure S8.** Graph showing the mortality % of brown planthopper adults **(A)** H_4_L and TBAPy solution at 500, 1000, 1500, 2000 mg/L; **(B)** silver nanoparticles at 5, 10, 15, 20 mg/L; The mortality data for DMSO or control treatments were less than 10%. Different letters above the bars of each figure indicate significant differences based on Duncan’s test at p≥ 0.05 between concentrations (mg/L) and different days. Each bar represents the mean ±SE of four replicates.


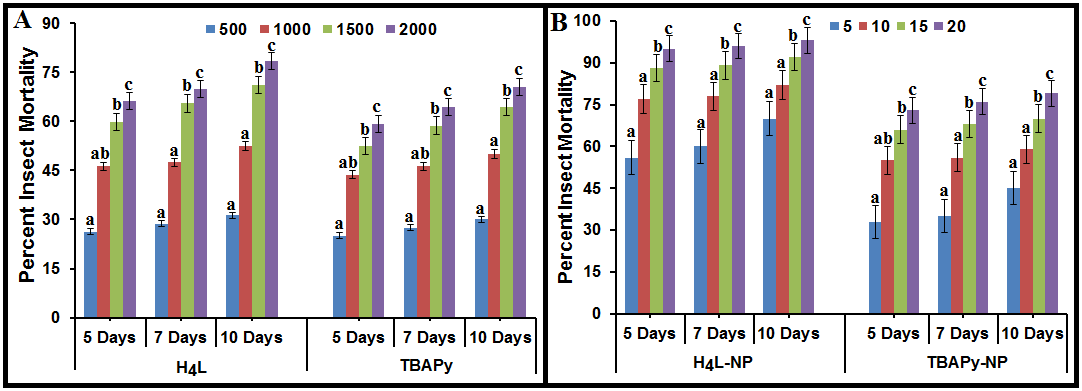

Supplement: Supplementary file 1 — Additional file 1. Scheme S1. [I] DMF, sodium carbonate, palladium acetate, 60 °C; [II] Extracted with ethyl acetate, Synthesis of 2’-Amino-1,1’:4’,1’’-terphenyl-3,3’’,5,5’’-tetracarboxylic acid (H4L). Scheme S2. Bromination and synthesis of 1,3,6,8-tetrakis (p-benzoic acid)pyrene (TBAPy). Figure S1. 1H NMR, for the 2’-amino-1,1’:4’,1’’-terphenyl-3,3’’,5,5’’-tetracarboxylate. Figure S2. 1H NMR, for the 1,3,6,8-tetrakis (4-(methoxycarbonyl)phenyl)-pyrene. Figure S3. 1H NMR, for the 1,3,6,8-tetrakis (p-benzoic acid)-pyrene. Figure S4. TEM images of the as-prepared H4L–AgNPs complexes with different AgNO3 concentrations. (A) 1 mmol, (B) 3 mmol, and (D) 5 mmol. Figure S5. (A, B) SEM image and elemental mappings of the as-obtained H4L–AgNPs illustrating the composition of the final product and the corresponding distribution of the observed Ag (C), Al (D), Cl (E), S (F), and O (G) components. (H) Plots of intensity versus the cross-sectional compositional line of the complex. Figure S6. EDX patterns of synthesized silver nanoparticles (A) H4L–AgNPs and (B) TBAPy–AgNPs. Figure S7. Physico‑chemical characterization of the nanoparticles. A and B: DLS profiles of the size distribution of H4L–AgNP and TBAPy–AgNP. C and D: Stability evaluation of Zeta potential analysis of H4L–AgNP and TBAPy–AgNP. Figure S8. Graph showing the mortality % of brown planthopper adults (A) H4L and TBAPy solution at 500, 1000, 1500, 2000 mg/L; (B) silver nanoparticles at 5, 10, 15, 20 mg/L; The mortality data for DMSO or control treatments were less than 10%. Different letters above the bars of each figure indicate significant differences based on Duncan’s test at p≥ 0.05 between concentrations (mg/L) and different days. Each bar represents the mean ±SE of four replicates. [file 12951_2021_1068_MOESM1_ESM.docx]
